# Anti-Swelling Polyelectrolyte Hydrogel with Submillimeter Lateral Confinement for Osmotic Energy Conversion

**DOI:** 10.1007/s40820-024-01577-0

**Published:** 2024-12-03

**Authors:** Yongxu Liu, Jiangnan Song, Zhen Liu, Jialin Chen, Dejuan Wang, Hui Zhi, Jiebin Tang, Yafang Zhang, Ningbo Li, Weijia Zhou, Meng An, Hong Liu, Guobin Xue

**Affiliations:** 1 Institute for Advanced Interdisciplinary Research (iAIR), School of Chemistry and Chemical Engineering, University of Jinan, Jinan, 250022 People’s Republic of China; 2 College of Mechanical and Electrical Engineering, Shaanxi University of Science and Technology, Xi’an, 710021 People’s Republic of China; 3 School of Physics and Technology, University of Jinan, Jinan, 250022 People’s Republic of China; 4 State Key Laboratory of Crystal Materials, Shandong University, 27 Shandanan Road, Jinan, 250100 People’s Republic of China

**Keywords:** Ionic polymer, Hydrogel, Confinement effect, Anti-swelling, Osmotic energy conversion

## Abstract

Harvesting the immense and renewable osmotic energy with reverse electrodialysis (RED) technology shows great promise in dealing with the ever-growing energy crisis. One key challenge is to improve the output power density with improved trade-off between membrane permeability and selectivity. Herein, polyelectrolyte hydrogels (channel width, 2.2 nm) with inherent high ion conductivity have been demonstrated to enable excellent selective ion transfer when confined in cylindrical anodized aluminum pore with lateral size even up to the submillimeter scale (radius, 0.1 mm). The membrane permeability of the anti-swelling hydrogel can also be further increased with cellulose nanofibers. With real seawater and river water, the output power density of a three-chamber cell on behalf of repeat unit of RED system can reach up to 8.99 W m^−2^ (per unit total membrane area), much better than state-of-the-art membranes. This work provides a new strategy for the preparation of polyelectrolyte hydrogel-based ion-selective membranes, owning broad application prospects in the fields of osmotic energy collection, electrodialysis, flow battery and so on.

**Supplementary Information:**

The online version contains supplementary material available at 10.1007/s40820-024-01577-0.

## Highlights

- Ionic polymers can directly serve as high-performance ion-selective membranes when it was physically confined within submillimeter-sized cylindrical pore.
- The universality of this strategy is demonstrated in preparing cation/anion-selective membrane.
- With real seawater and river water, the output power density of a three-chamber cell on behalf of repeat unit of reverse electrodialysis system can reach up to 8.99 W m^−2^.

**Supplementary Information:**

The online version contains supplementary material available at 10.1007/s40820-024-01577-0.

## Graphical abstract

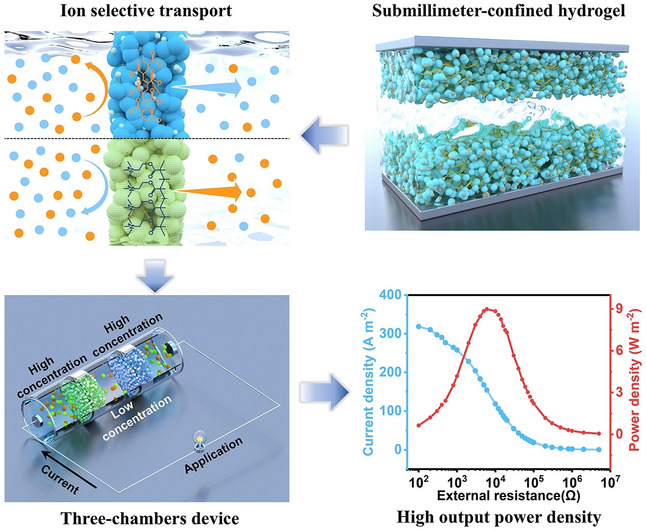

## Introduction

The development of sustainable energy sources, such as solar, wind, geothermal and other clean energy, is urgent to combat the energy crisis [1–3]. Among various renewable energy sources, the earth-abundant salt difference energy is exactly promising [4, 5]. In theory, the total amount of salinity gradient energy from the confluence of rivers and oceans can reach about 1.4–2.6 TW, which is close to the global electricity demand of about 2.8 TW in 2020 [6]. The salinity gradient energy [7] can be directly converted into electricity energy with membrane-based [8] reverse electrodialysis (RED) technology [9–11]. The maximum energy conversion efficiency can be high as 100% when anion and cation were driven by salinity gradient to separately pass through anion- and cation-selective membrane [12, 13]. During the energy conversion process, the conversion efficiency is highly affected by the ion selectivity of the membrane [14–16]. On the other hand, the converted electricity will be dissipated by the internal resistance of the membrane which lows the output power density [17–19]. Therefore, it is particularly important to optimize the membrane permeability and ion selectivity to achieve high energy conversion efficiency and output power density [13, 19]. Usually, the ion selectivity will decrease when the membrane permeability increases. Simultaneously achieving excellent ion selectivity and membrane permeability is still one of the main challenges to prepare high-performance ion-selective membrane [10, 20, 21].

Pore size at the level of Debye length and high surface charge density are two essential parameters to achieve excellent ion transport selectivity [22]. Ionic polymer is one of the earliest candidates to prepare ion-selective membrane owing to its low cost, charged 3D network structures and excellent ion transport capacity [23]. However, the ionic polymer is easy to swelling because of the strong water-absorbing capacity and weak polymer skeleton structure. In traditional heterogeneous ion-selective membranes, ionic polymer and resin is mixed together to inhibit the swelling of ionic polymer and ensure the special pore size for ion-selective transport [24]. The resin will increase the ionic resistance of the composite in plain sight. So low-cost heterogeneous ion-selective membranes mainly hold its own in the primary step of electrodialysis process [25]. Because of the low cost and easy preparation, efforts toward practical ionic polymer-based ion-selective membranes are never stopped [2, 26, 27]. For example, there have been reports of introducing sulfonated functional groups [28], fluorinated functional groups [23, 29] or electrostatic bonding of dissimilar charged functional groups into hydrogels to resist swelling [30]. Nanoscale confinement effect was also used to inhibit the swelling of ionic polymer hydrogel [31, 32]. However, these methods have some problems, such as high preparation cost, complex processing technology and toxic materials [14].

Herein, we demonstrate that physically confined ionic polymer itself can be a high-performance ion-selective membrane. We construct macroscopical pore in aluminum sheet with laser and further anodized of the aluminum sheet to be positive or negative charged. Sodium polyacrylate (PAAS) and methylacryloxyethyl trimethylammonium chloride (DMC) are selected as typical ionic polymer and confined in pore of aluminum sheet with electrostatic bonding. We find that even the lateral size of the pore up to the submillimeter scale, the ionic polymer hydrogel can still present excellent ion selectivity, which was attributed to the anti-swelling ability in physically restricted conditions. We also show that cellulose nanofibers (CNFC) can further increase the ion permeability of the ionic polymer hydrogel. Thus, we get ionic polymer-based ion-selective membrane which remains its excellent ionic transport capacity. When one pair of cation/anion-selective membrane is used in RED system, the output power density can reach 8.99 W m^−2^. This opens up the way for preparing low-cost ionic polymer-based membranes to realize efficient permeation energy conversion.

## Experimental Procedures

### Materials and Chemicals

Acrylic acid (AA, > 99%) was purchased from Aladdin. Ammonium persulfate ((NH_4_)_2_S_2_O_8_) was purchased from Aladdin. N, N’-Methylenebis(acrylamide) (C_7_H_10_N_2_O_2_) was purchased from Macklin. [2-(Methacryloyloxy)ethy] trimethylammonium chloride solution and cotton nanofiber cellulose (CNFC) were purchased from Qihong, oxalic acid was purchased from Aladdin, sodium tetraborate was purchased from Leyan. Sodium hydroxide (NaOH), sodium chloride (NaCl), potassium chloride (KCl) and lithium chloride (LiCl) were purchased from Ron reagent. All chemicals are analytically pure and were used as without any further purification.

### Fabrication of CNFC-PAAS Hydrogel and CNFC-DMC Hydrogel

CNFC solution (1 wt%) and AA monomer were firstly mixed, and then, 10 M NaOH solution was added to the above solution to neutralize AA monomer in an ice bath under vigorous stirring. After stirring for 30 min, ammonium persulfate (APS) initiator and N, N’ -methylene bis (acrylamide) (MBAA) crosslinking agent were successively added. The homogeneous and transparent precursor solution of fibrous sodium polyacrylate (PAAS) was then heated at 60 °C for 3 h to prepare a 1-mm-thick polymer hydrogel film.

Similarly, 1 wt% CNFC solution and 75 wt% DMC (10 mL) solution were uniformly mixed and then 9 mg of ammonium persulfate (APS) initiator, 3.1 mg of N, N’-methylenebisacrylamide (MBAA) crosslinker, and 1 μL of N, N, N’, N’-tetramethylethylenediamine (TEMED) promoter in sequence. After stirring for 30 min, the uniform fibrous precursor solution of acryloyloxyethyltrimethyl ammonium chloride water gel (DMC) is obtained. The reaction solution was then reacted in a 60 °C vacuum oven for 3 h, resulting in a 1-mm-thick polymer hydrogel film.

### Fabrication of CNFC-PAAS-AAS Membrane and CNFC-DMC-AAS Membrane

Pure Al (#1060, 99.7%, 0.2 mm in thickness) plate was first ultrasonically cleaned with acetone, ethanol and distilled water in turn for 15 min. Laser boring was constructed with laser marker (HTF20T, Hanser, China) equipped with a fiber laser (Reycus, China) with a wavelength of 1064 nm. Then anodization was carried to prepare p/n-charged AAS. For n-AAS, aluminum sheet served as the anode and was immersed in 0.3 mol L^−1^ oxalic acid solution at 90 °C. The anodization process was constructed with constant voltage of 40 V for a duration of 2 h. For p-AAS, aluminum sheet served as the anode and was immersed in 0.3 mol L^−1^ sodium tetraborate solution at 90 °C. The anodization process was also constructed with constant voltage of 40 V for a duration of 2 h. The anodic aluminum oxide was then fixed at the bottom of a culture dish and vacuum soaked in the hydrogel precursor solution for 10 min. Finally, the culture dish was placed in a 60 °C oven for 30 min to realize polymerization reactions. To ensure the strong surface bonding interaction, PAAS hydrogel was adhered with p-AAS and DMC hydrogel was adhered with n-AAS.

### Characterization

The scanning electron microscopy (SEM) images were obtained with a Hitachi Regulus-8100 Field Emission Scanning Electron Microscope. The Zeta potentials testing was measured by using Zetasizer Nano ZS. The Fourier transform infrared (FT-IR) spectroscopy was measured by using VERTEX 70 Spectrometer.

### Electrical Measurements

The electrochemical testing was constructed with an electrochemical workstation (CHI760E B18569). A pair of home-made Ag/AgCl electrodes were used to apply the transmembrane potential. Cyclic voltammetry was used to record the *I-V* curve. The range of the sweeping voltage was from − 0.3 to + 0.3 V, and the step voltage was 0.001 V. Except for the time test, all measurements were completed within 30 min.

### Molecular Dynamics Simulations

The swelling rate of bulk and confined hydrogels was studied by molecular dynamics (MD) simulations by the large-scale atomic/molecular massively parallel simulator (LAMMPS) package. The consistent valence force field (CVFF) force field is used to describe the bond interaction among atoms of PAAS molecular chains. TIP4P water model is adopted to design our simulations. The nonbonded interactions among ions, water molecules and PAAS chains were described by Lennard–Jones 12–6 potential. The periodic boundary conditions were applied along all the directions. The cutoff radius for long-range interaction calculation was set to be 10 Å. The velocity Verlet algorithm with time step 0.25 fs was used to integrate Newton’s equations of motion. The long-range interaction was calculated via the particle–particle particle-mesh (PPPM) approach with error parameter of 10^−4^.

For the swelling of bulk hydrogel, four simulation models of hydrogels with water content of 26, 64.4, 68.1 and 68.9 wt% were constructed, consistent with experimental samples. After energy minimization, NPT ensemble with a constant atom number, pressure and temperature, is performed and the system temperature was heated from 300 to 600 K to fully relax the simulation system for 0.5 ns. Then, another NPT ensemble is performed to realize the annealing process with temperature from 600 to 300 K for 0.5 ns. Furthermore, one NVT-MD ensemble was performed at 300 K for 0.1 ns. Finally, the fully swelled bulk hydrogel samples with various water contents were obtained. Their simulation cells are 140.6 nm^3^ for 26 wt%, 328.5 nm^3^ for 64.4 wt%, 373.2 nm^3^ for 68.1 wt%, 405.2 nm^3^ for 68.9 wt%.

As for the swelling of confined hydrogels, the built simulation model is shown in Fig. 5, where the confined space is constructed by removing aluminum atoms in the pore and PAAS hydrogel was uniformly placed in the simulation cell. Each sample was equilibrated via NPT-MD simulations at 300 K and 1 atm until the temperature and density of simulation system do not change with simulation time. Then, another further 0.5 ns simulation was performed in the NVT-MD. The obtained system size is 6.88 × 6.88 × 19.7 nm^3^, and the dimensional length of nanopore is 2.75 × 2.75 × 2.75 nm^3^. The data were recorded in the NVE-MD for 10 ns.

## Results and Discussion

### Construction and Characterization of CNFC-PAAS-AAS

**Fig. 1:**
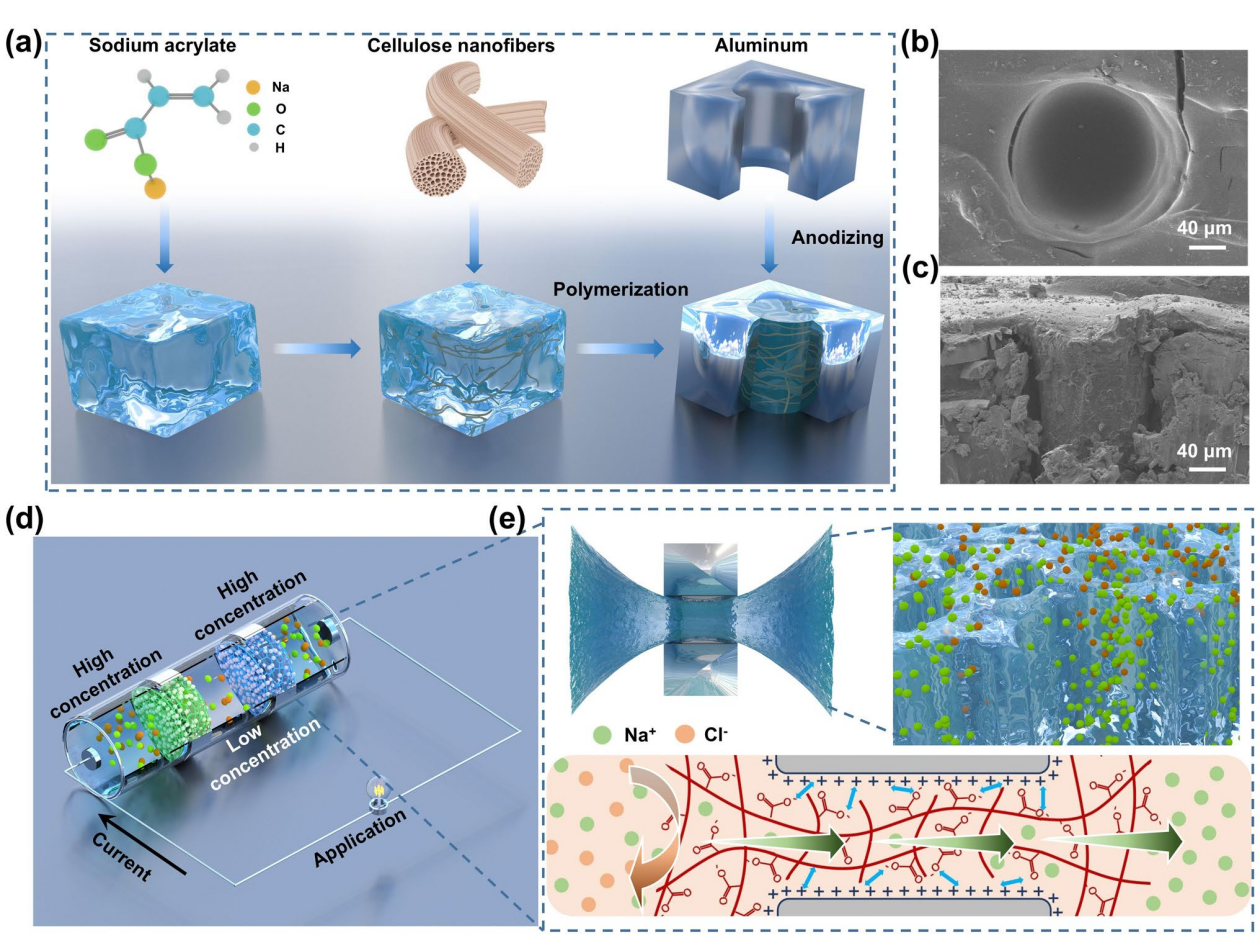
Preparation and characterization of physically-confined hydrogel with submillimeter pore. **a** Schematic diagram of the preparation of CNFC-PAAS-AAS membrane. Macroscopical pore is constructed in aluminium sheet with laser and further anodized to be positively charged. The precursor of sodium polyacrylate and cellulose nanofibersare mixed and then polymerized in the pore. **b** Top and **c** cross-sectional views of the CNFC/PAAS hydrogel confined in the anodized aluminium substrate (AAS) pore. **d** Schematic illustration of CNFC-PAAS-AAS and CNFC-DMC-AAS membranes used in osmotic energy conversion. **e** PAAS hydrogel is taken as the example to show the origin of ion selectivity. The swelling of PAAS hydrogel at the perpendicular direction was inhibited by the hard wall. Thus the swelling of the network hydrogel at the radial direction was also inhibited by the entangled molecular chain. The confined nanochannel in the PAAS hydrogel will favor the transport of counter-ions (Na^+^) from the high-concentration reservoir (left) to the low-concentration reservoir (right)

Figure 1a shows the preparation process of PAAS hydrogel-based cation-selective membrane. Aluminum sheet with a thickness of 0.2 mm is used as the matrix and millimeter-sized pore can be easily constructed with laser machining technology (Fig. S1). Since PAAS is negatively charged, anodic oxidation is adopted to prepare negatively charged alumina at the surface of the aluminum sheet [33]. It is reported CNFC can enhance the ionic conductivity of the ionic polymer hydrogel [34–37]. So CNFC is also adopted here to enhance the membrane permeability, which can be easily achieved by mixing the CNFC with the acrylic acid monomer during the polymerization process (Fig. S3). Figure 1b and c are the SEM images of the PAAS hydrogel confined in the cylindrical submicron pore. It can be seen that the submillimeter pore was well filled with the hydrogel. Except PAAS, DMC is adopted as a typical anionic polymer to prepare anion-selective membrane. When compositing DMC and aluminum sheet, the aluminum sheet is negatively charged to ensure the binding force [33]. We found that when the polymer hydrogel was physically confined within cylindrical pore, it can serve as high-performance ion-selective membranes for osmotic energy harvesting (Fig. 1d). As shown in Fig. 1e, when the PAAS hydrogel is confined in the cylindrical pore, the swelling at the perpendicular direction is inhibited by the hard wall. So, the hydrogel can only swell at the radial direction. The entangled molecular chain will also inhibit the swelling at the radial direction since the point at the wall is fixed. The channel size in the confined hydrogel can maintain at the level of Debye length. Together with abundant anionic carboxylic, the PAAS hydrogel can effectively reject Cl^−^ ions and only Na^+^ ions are favorably transported under the salinity gradient. It is amazing that the ionic polymer hydrogel can maintain the ion selectivity when the radius controlling up to submillimeter scale. Submillimeter-sized pore can be easily fabricated with current state of the art, such as mechanical microdrilling technology and pulse laser machining technology (Fig. S3). Together with the low cost of ionic polymer, the proposed membrane preparation technique will promote the development of low-cost ion-selective membrane.

**Fig. 2:**
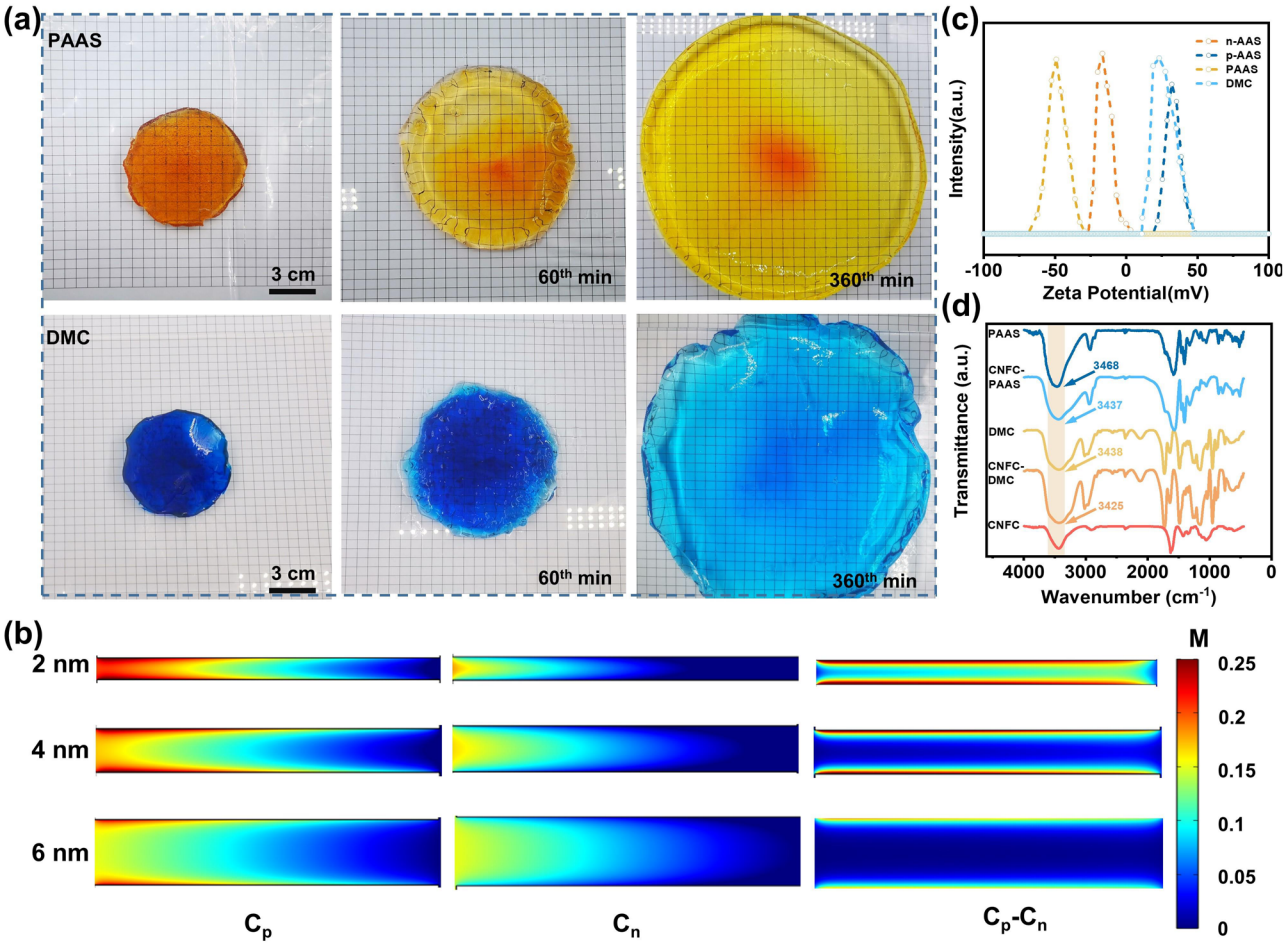
Characterization of the oppositely charged hydrogel. **a** The free swelling process of PAAS and DMC hydrogel in water. **b** Numerical simulation of the ionic concentration of C_p_ (concentration of Na^+^ ions), C_n_ (concentration of Cl^–^ ions) and C_p_-C_n_ in different nanochannel. **c** Zeta potential of positively/negatively charged AAS (p-AAS, n-AAS), PAAS and DMC. **d** FT-IR spectra of PAAS, DMC, PAAS/CNFC and DMC/PAAS

Figures 2a and S4 show the free swelling process of the PAAS and DMC hydrogel in water and salt solution, respectively. The hydrogels are dyed with orange and blue color for clarify. The diameter of hydrogel increased about two times in 60 min, and it is easy to think that the ion transport channel will expand when the hydrogel is swelling. The size of channel is essential to the ion selectivity. Only when the size of the channel is at the level of Debye length, the ion transport can be effectively governed by the surface charge. The size of nanochannels in PAAS hydrogel is calculated to be 2.4 nm (Supplementary Information). As shown in Fig. 2b, we studied the ion transport in the nanochannel with different diameter with numerical simulation (Figs. S5, S6). The positively charge Na^+^ will be attracted and the negatively charged Cl^−^ will be repelled by the negatively charged channel, respectively. The concentration difference of these two ions represents the ion selectivity of the nanochannel. It is obvious that the ion selectivity in 6-nm channel is poorer compared to 2 nm channel. It can be derived backward that the hydrogel is anti-swelling when it maintains the ion selectivity. To assure the tight bind between the hydrogel and aluminum matrix, charged anodic oxide film was prepared on aluminum by electroplating (Fig. S7). The zeta-potential of ionic polymer and alumina are shown in Fig. 2c. PAAS and DMC present negative and positive zeta potential, respectively, because of their ionizable functional groups. Because of the small content (1 wt%), the zeta potential of CNFC-PAAS and CNFC-DMC is very similar to PAAS and DMC, respectively (Fig. S8). The Al substrate is also charged because of the adsorption of H^+^ or OH^−^ during anodic oxidation process. FT-IR is used to confirm the binding activity between cellulose and PAAS chains [38, 39]. Comparing with the O–H absorption peak of PAAS hydrogel at 3468 cm^−1^, a red shift occurs in CNFC-PAAS (about 3437 cm^−1^). Similarly, CNFC-DMC also generates a red shift (about 3425 cm^−1^) compared to DMC (about 3438 cm^−1^), indicating the formation of hydrogen bond between CNFC and PAAS (Fig. 2d) [40, 41].

### Transmembrane Ion Transport

**Fig. 3:**
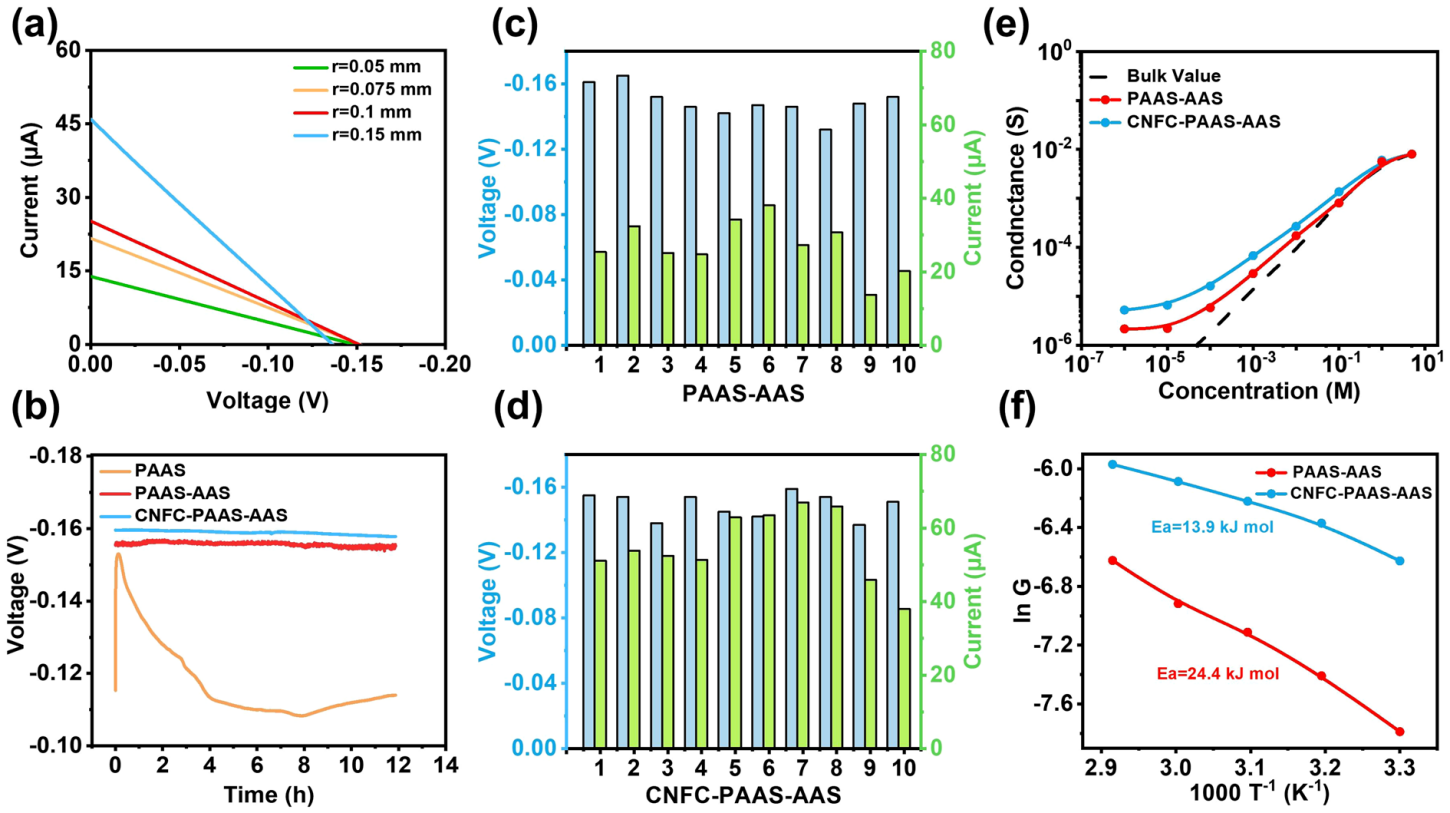
Transmembrane ion transport properties. **a** *I-V* curves of PAAS hydrogel confined in AAS pore with different lateral sizes. **b** *V-t* curves of the CNFC-PAAS-AAS, PAAS-AAS and the PAAS hydrogel. **c** Open-circuit voltage (*V*_OC_) and short-circuit current (*I*_SC_) of ten PAAS-AAS membranes. **d** *V*_OC_ and *I*_SC_ of ten CNFC-PAAS-AAS membranes. **e** Ionic conductivity of PAAS-AAS and CNFC-PAAS-AAS at different NaCl concentrations. **f** The Arrhenius-type plot of the ion conductivity variation with temperature. The Na^+^ ion transmembrane barrier was 13.9 kJ mol^−1^ and 24.4 kJ mol^−1^ under a 50-fold salinity gradient in CNFC-PAAS-AAS and PAAS-AAS, respectively

We first confirm the maximum pore size with which the pore could effectively inhibit the swelling of ionic polymer hydrogel. The membrane is installed in a two-chamber device with different salinity, and the anti-swelling ability is evaluated by the ion selectivity of the membrane which is associated with the open-circuit voltage (*V*_OC_) [42]. As shown in Fig. 3a, the *V*_OC_ of PAAS-AAS membrane with different pore size is measured. When the radius of the pore is smaller than 0.1 mm, *V*_OC_ is about 0.15 V, which denotes the excellent ion selectivity of the membrane [43]. When the radius of the pore exceeds 0.1 mm, the open-circuit voltage obviously decreases, indicating that the ion selectivity is getting worse. The bigger the pore size, the easier the pore is to be fabricated using mechanical micro-drilling or pulse laser machining technologies. Thus, we chose the pore with the radius of 0.1 mm to inhibit the swelling of the ionic polymer hydrogel. In general, cellulose nanofibers containing rich functional groups can greatly improve the ion transport capacity based on the jump effect [34]. Therefore, cellulose nanofiber is adopted here to enhance the ionic conductivity of the PAAS hydrogel and thus improve the output power density (Fig. S9). Figure 3b shows the open-circuit voltage of free PAAS hydrogel, confined PAAS hydrogel and CNFC-PAAS hydrogel in a long term. It can be seen that the open-circuit voltage of free PAAS hydrogel rapidly decreases to 0.11 V because of the swelling. The open-circuit voltage of confined PAAS hydrogel and CNFC-PAAS hydrogel can remain about 0.16 V at 50-fold concentration gradient in 12 h, demonstrating the excellent stability. Then membranes of confined PAAS hydrogel and CNFC-PAAS hydrogel are tested with the radius of 0.1 mm as shown in Fig. 3c and d. The similar values of open-circuit voltage and short-circuit current indicate the stability of these membranes. It can be seen that after mixing CNFC, the open-circuit voltage of the membrane slightly increases, while the short-circuit current improves obviously. The open-circuit voltage of CNFC-PAAS-AAS membrane is also affected by the radius of the pore (Fig. S10). During the 12-h *V-t* test, the membrane with a radius of 0.1 mm exhibits the best stability (Fig. S11). When the thickness of the membrane is added, the membrane resistance will increase and thus the output power density will decrease (Fig. S12). It should be noted that with neutral aluminum substrate (without anodic oxidation process), the film also presents good ion selectivity and similar membrane permeability (Fig. S13). The ionic conductance in a range of NaCl concentrations is plotted as a function of electrolyte concentration (Fig. 3e). When the concentration of NaCl solution is lower than 1 M, the ionic conductance of CNFC-PAAS-AAS deviates significantly from the bulk value (black dashed line), indicating that the ion transport behavior across the membrane is controlled by the surface charge. Meanwhile, the conductance of CNFC-PAAS-AAS is much higher than that of PAAS-AAS membrane, which indicates that CNFC-PAAS-AAS membrane is more suitable for energy harvesting. Based on the thermodynamic process, the ion transport energy barrier in the 2D nanofluidics could be calculated according to the Arrhenius equation [44]:

1 $$G={G}_{0}{e}^{-\frac{{E}_{a}}{RT}}$$

where *G* represents the ion conductance, *G*_0_ is an Arrhenius constant, *E*_a_ represents the activation energy of ion transport, *R* and *T* denote the gas constant and temperature, respectively. According to the calculation, the *E*_a_ of CNFC-PAAS-AAS membrane is 13.9 kJ mol^−1^, which is obviously lower than that of PAAS-AAS membrane (24.4 kJ mol^−1^) (Fig. 3f). This indicates that CNFC can effectively lower the energy barrier of ion transport in PAAS-AAS membrane.

### Osmotic Energy Conversion

**Fig. 4:**
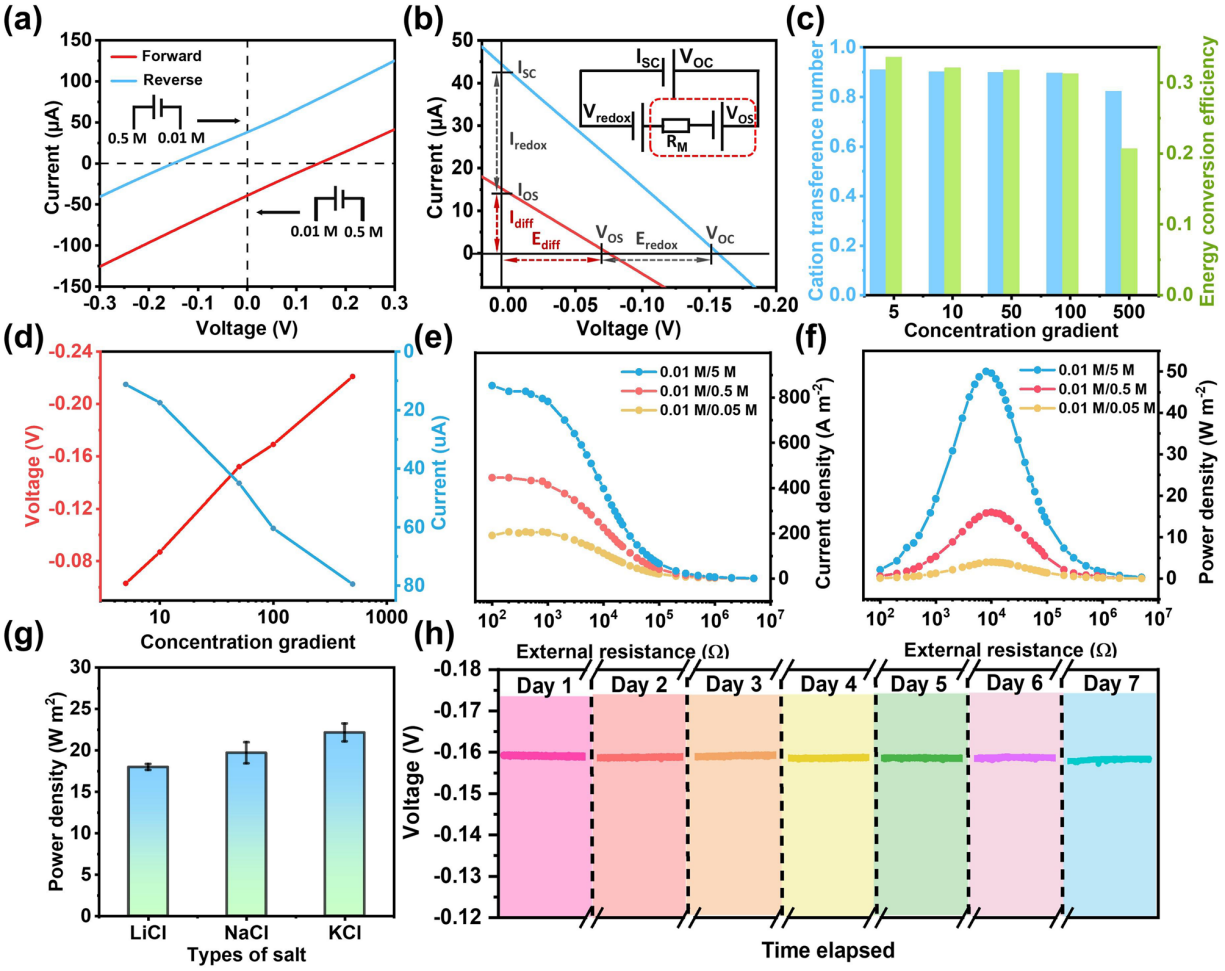
Osmotic energy conversion with single CNFC-PAAS-AAS membrane. **a** *I-V* curves of the CNFC-PAAS-AAS at 50-fold NaCl concentration gradient under forward and reverse diffusion directions. **b** *I-V* curve (blue solid line) of the membrane at 50-fold concentration gradient. The contribution of the redox potential generated at the electrode is subtracted to obtain the osmotic voltage and osmotic current (red line). The inset shows the equivalent circuit of the osmotic energy harvesting system. **c** The cation transfer number and the energy conversion efficiency under different concentration gradients. **d** Open-circuit voltage (*V*_OC_) and short-circuit current (*I*_sc_) as a function of the salt gradient for the CNFC-PAAS-AAS membrane. **e** Current density and **f** output power density of the CNFC-PAAS-AAS membrane with different external resistance under three different salinity gradients. **g** Maximum output power density of the CNFC-PAAS-AAS membrane with different electrolytes at 50-fold concentration gradient. **h** Open-circuit voltage of CNFC-PAAS-AAS membrane in a continuous 7 days testing

Figure 4a shows the representative *I-V* curves of the CNFC-PAAS-AAS membrane at 50-fold concentration gradient (0.5/0.01 M NaCl solution, Fig. S14). Under the reverse concentration gradient, the absolute values of short-circuit current and open-circuit voltage are similar to those under the forward concentration gradient, but they have different polarities due to the opposite direction of ion diffusion. This indicates that the membrane is symmetric and ion diffusion has no preferential direction. The osmotic potential (*V*_diff_) of the nanofluid membrane can be obtained by subtracting the redox potential (*V*_redox_) at the electrode/solution interface from the measured *V*_OC_ (Figs. 4b and S15) [14, 45–54]. Figure 4c shows the cation transfer number (*t*_+_) and energy conversion efficiency (*η*) under different concentration gradients (Table S1). The NaCl concentration is set to 0.01 M at low concentration side and increased from 0.1 to 5 M at high concentration side. At 50-fold (0.5/0.01 M) concentration gradient, the ion transfer number and energy conversion efficiency of CNFC-PAAS-AAS membrane are calculated to be 0.899% and 31.8%, respectively (Table S1). It can be seen that both of them reach the maximum value at 50-fold concentration gradient. In addition, the *V*_OC_ and *I*_SC_ under a series of concentration gradients are recorded and both increased with the concentration gradient (Fig. 4d). The maximum output power density can be obtained when the internal resistance of the membrane is equal to the load resistance. As shown in Fig. 4e, under different salinity gradients, the current density on the external circuit decreases as the resistance value of the load resistance increases (calculated with the area of the hydrogel contained within the aluminum pore). When the load resistance is about 8 kΩ, the maximum output power density is 16.04 W m^−2^ at 50-fold concentration gradient; compared with the membrane in the same test area, CNFC-PAAS-AAS has excellent power density (Fig. S16). In addition, the maximum output power densities are 3.99 and 50.02 W m^−2^ at 5-fold and 500-fold concentration gradient, respectively (Fig. 4f and Table S2). It should be noted here that the contribution of electrode potential is not subtracted here. The energy conversion behaviors of the membranes are also highly associated with the type of electrolytes. We tested the power density of the membrane under three different salt solutions with concentration gradients of 0.5/0.01 M. As shown in Fig. 4g, the system achieves the maximum power output for the KCl (21.6 W m^−2^) and the minimum power output for the LiCl (15.4 W m^−2^), because the ion diffusion coefficient order is K^+^ > Na^+^ > Li^+^. In addition, since the long-term stability strongly influenced the real-world application in osmotic power harvesting, we chose a membrane with excellent ion selectivity (high open-circuit voltage) to perform long-term experiment. As shown in Fig. 4h, the CNFC-PAAS-AAS membrane only lost 1.26% of its origin performance over 7 days of continuous testing. In a two-week continuous testing, the short-circuit current and open-circuit voltage are both stable, confirming the excellent stability of the membranes in osmotic energy conversion (Fig. S17). Higher temperature will obviously enhance the short-circuit current and thus the output power density. The membrane also shows excellent stability at 343 K (Fig. S18).

### Molecular Dynamics Simulations

**Fig. 5:**
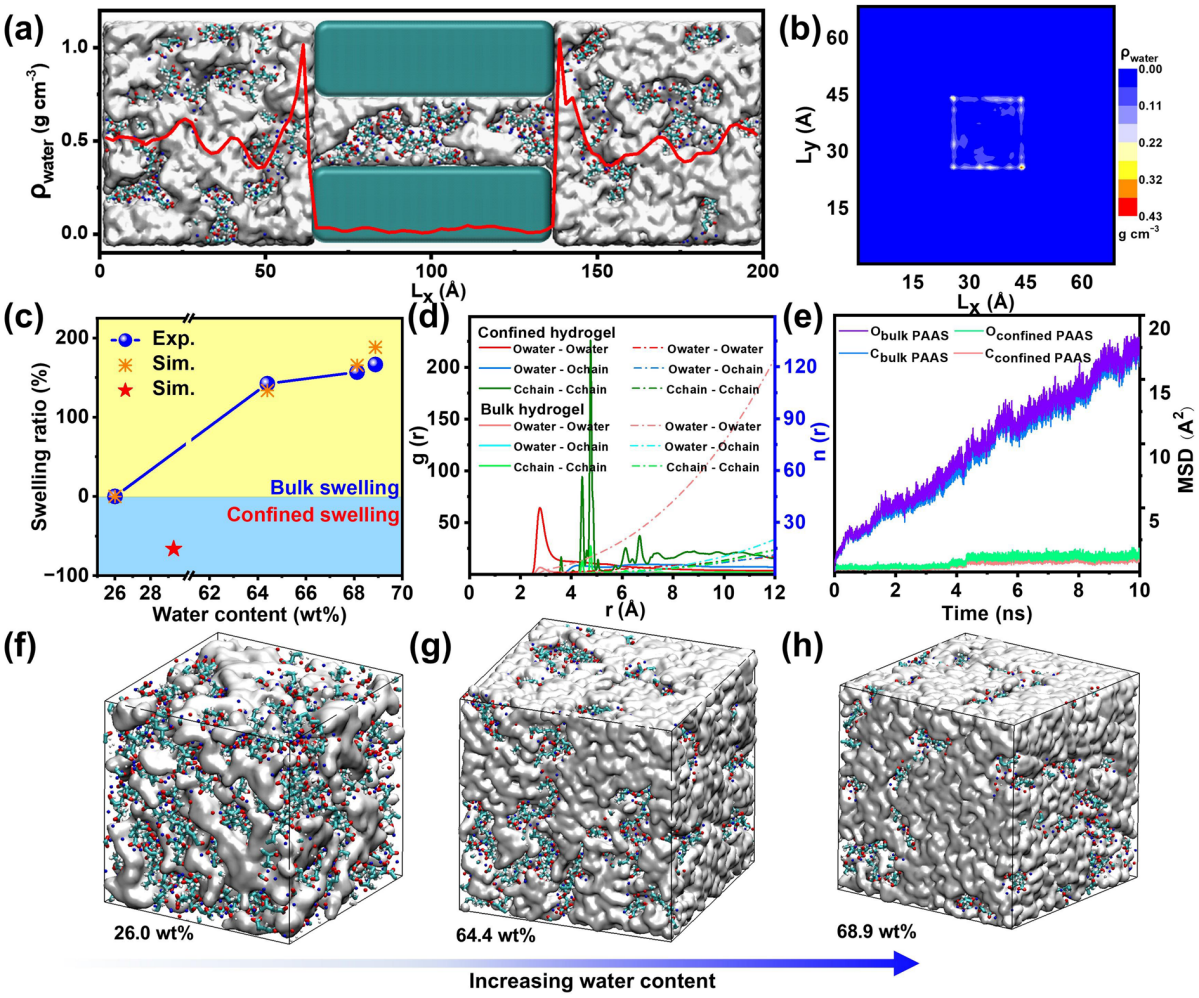
Molecular dynamics simulations of the swelling process in physically-confined ionic polymer hydrogel. **a** The MD snapshot of confined ionic polymer hydrogel, where the inserted curve is the mass density of water molecules. **b** The mass density mapping in x-y plane. **c** Swelling ratio of bulk and confined ionic polymer hydrogels with different water content. **d** Radial distribution function (RDF) and coordination number of atom pairs Owater-Owater, Owater-OPAAS, CPAAS-CPAAS in confined and bulk ionic polymer hydrogels. **e** The mean square displacement (MSD) of carbon and oxygen atoms in confined and bulk ionic polymer hydrogels. **f**–**h** The MD snapshots of bulk ionic polymer hydrogels with water content of 26.0 wt%, 64.4 wt% and 68.9 wt%

To elaborate the swelling mechanism of ionic polymer hydrogels in confined aluminum pore, the simulation system of confined PAAS hydrogel is constructed as shown in Fig. 5a. Periodic boundary condition is applied in three directions of simulation cell. The PAAS hydrogel is uniformed placed in the confined space of aluminum pore and free space at two sides. It is obviously found that the water density in confined pore is much smaller than that of bulk counterpart. To further visualize the spatial distribution of water molecules inside aluminum pore, the mass density mapping in *x*–*y* plane is shown in Fig. 5b. It is observed that almost all the water molecules are arranged in the surface of aluminum pore. In other words, the finite-sized pore restricts the permeation of water molecules into PAAS polymer network, further limiting the swelling of PAAS hydrogel. To quantitatively characterize the confined swelling ratio of PAAS hydrogel, another three bulk hydrogel samples in Fig. 5f-h with water content of 26, 64.4 and 68.9 wt% are constructed and the corresponding swelling ratios are calculated, as shown in Fig. 5c. Notably, the swelling ratio of bulk hydrogel increases with the increasing water content. Interestingly, the value of swelling ratio is negative for confined PAAS hydrogel, indicating the water content of confined hydrogel is much smaller than that of bulk hydrogel. Moreover, mean square displacement (MSD) of carbon and oxygen atoms of PAAS chains in confined pore and free spaces are counted in Fig. 5e. Compared with the diffusion of PAAS chains in free spaces, the carbon and oxygen atoms of PAAS chains are severely suppressed, indicating the polymer network of PAAS chains is stable and beneficial to realizing a stable ion selectivity of hydrogel. Furthermore, the radial functions (RDF) of O_water_-O_water_, O_water_-C_PAAS_, C_PAAS_-C_PAAS_, defined as the probability of finding one atom at a certain distance from another tagged atom, are calculated for water molecules and PAAS chain in the confined pore and free space. Obviously, the RDF peak amplitude of O_water_-O_water_ in confined space is much larger than that of free space, indicating the water molecules are arranged in specified positions. Moreover, compared with bulk hydrogel, more RDF peaks of C_PAAS_-C_PAAS_ in confined pore are observed, indicating the more ordered arrangement of PAAS chains in the pore. Therefore, the confined space of aluminum pore enables the redistributed water molecules and PAAS chains in pore space and effectively restricts the swelling of PAAS hydrogel, which facilitates the long-term high-performance ion selectivity of confined ionic polymer hydrogels.

### Application of RED

**Fig. 6:**
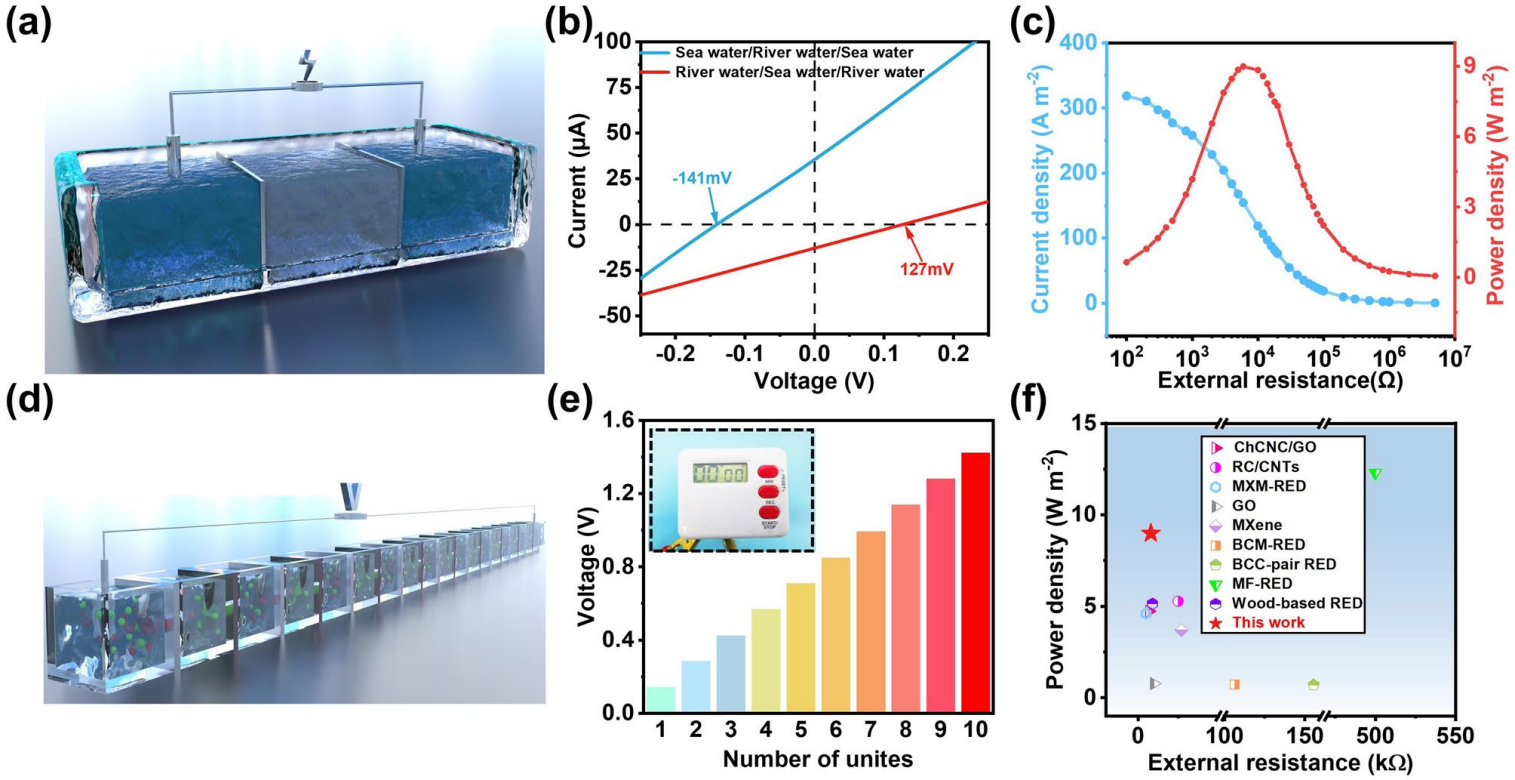
Osmotic energy conversion with a pair of cation/anion selective membrane. **a** Three-chamber electrochemical cell with a pair of CNFC-PAAS-AAS and CNFC-DMC-AAS membrane. **b** The *I-V* curves of the three-chamber cell under two configuration methods of central high concentration and central low concentration. **c** Current density and output power density with real river water and seawater. **d** Schematic illustration of the tandem stacks of RED device with alternant CNFC-PAAS-AAS and CNFC-DMC-AAS membrane. **e** The voltage of the RED systems varies with the number of cell units. **f** Power generation performance of the three-chamber cell (red star) compared with state-of-the-art osmotic power generators

Anion-selective membrane was also constructed with DMC hydrogel (Fig. S19 and Table S3). A three-chamber on behalf of repeat unit of RED system was used to demonstrate the scalability of this physically confined strategy (Fig. 6a) [55]. Anion-selective membrane CNFC-DMC-AAS and cation-selective membrane CNFC-PAAS-AAS are assembled into the three-chamber cell (Fig. S20). Two representative *I-V* curves (Fig. 6b) are obtained by testing at two different concentration configurations of HLH (seawater/river water/sea water) and LHL (river water/sea water/river water) [56]. Considering that *V*_OC_ and *I*_sc_ produced by HLH are higher than LHL, HLH is regarded as the focus of research. When tested in real river seawater, the system achieved a maximum output power density of 8.99 W m^−2^ with an external resistance of 8 kΩ (Fig. 6c and Table S4). When 10 series units were connected, the open-circuit voltage can reach up to 1.42 V, demonstrating the excellent osmotic energy conversion performance (Fig. 6d) [57]. In addition, a good linear relationship between voltage and the number of RED devices can be observed, indicating that RED devices have good consistency and stability (Figs. 6e and S21). Comparing with the state-of-the-art osmotic energy conversion systems, the output power density of our RED system is among the best value ever reported, demonstrating the advantage of the ion-selective membrane constructed with the proposed physically confined strategy (Fig. 6f and Table S5) [52, 58–65].

## Conclusion

In summary, we propose that submillimeter-sized pore can effectively inhibit the swelling of ionic polymer hydrogel and the confined hydrogel can work excellently as ion-selective membrane to harvest osmosis energy. We demonstrate the properties of cation-selective membrane with confined PAAS hydrogel and CNFC can be used to further improve the ionic conductivity of the hydrogel. The ion selectivity of the single cation-selective membrane can be up to 0.899 at 50-fold salinity gradient. We also prepare anion-selective membrane with DMC hydrogel and thus RED device with a pair of cation/anion-selective membrane are constructed with the output power density up to 8.99 W m^−2^. This study provides a strategy for preparing high-performance ion-selective membrane with physically confined ionic polymer hydrogel and reveals a new chapter in porous material design.

## Supplementary Information

Below is the link to the electronic supplementary material.

Supplementary file1 (DOCX 22320 kb)
