# Supplementary material for: Anti-Swelling Polyelectrolyte Hydrogel with Submillimeter Lateral Confinement for Osmotic Energy Conversion
Source: Nanomicro Lett. 2024 Dec 3;17:81. doi: 10.1007/s40820-024-01577-0 (PMC11612061; doi:10.1007/s40820-024-01577-0)
Supplement: Supplementary file 1 — Supplementary file1 (DOCX 22320 kb) [file 40820_2024_1577_MOESM1_ESM.docx]

Supporting Information for

**Anti-Swelling Polyelectrolyte Hydrogel with Submillimeter Lateral Confinement for Osmotic Energy Conversion**

Yongxu Liu ^1,#^, Jiangnan Song ^2,#^, Zhen Liu ^1^, Jialin Chen ^1^, Dejuan Wang ^1^, Hui Zhi ^1^, Jiebin Tang ^1^, Yafang Zhang ^3^, Ningbo Li ^1^, Weijia Zhou ^1^, Meng An ^2,^*, Hong Liu ^1,4,^*, Guobin Xue ^1,^*

^1^ Institute for Advanced Interdisciplinary Research (iAIR), School of Chemistry and Chemical Engineering, University of Jinan, Jinan, 250022, P. R. China

^2^ College of Mechanical and Electrical Engineering, Shaanxi University of Science and Technology, Xi'an 710021, P. R. China

^3^ School of Physics and Technology, University of Jinan, Jinan, 250022, P. R. China

^4^ State Key Laboratory of Crystal Materials, Shandong University, 27 Shandanan Road, Jinan, 250100, P. R. China

^#^Yongxu Liu and Jiangnan Song contributed equally to this work.

* Corresponding authors. E-mail: [ifc_xuegb@ujn.edu.cn](mailto:ifc_xuegb@ujn.edu.cn) (Guobin Xue); [anmeng@sust.edu.cn](mailto:anmeng@sust.edu.cn) (Meng An); [hongliu@sdu.edu.cn](mailto:hongliu@sdu.edu.cn) **(**Hong Liu)

## S1 Calculation of energy conversion efficiency

From the equivalent circuit diagram in the power generation system (Figs. 4b and S22), the measured open-circuit voltage (*V*_OC_) actually consists of the diffusion potential (*V*_diff_) and redox potential (*V*_redox_) [S1-S3]：

$$V_{OC}=V_{diff}+V_{redox} \left( S1 \right)$$

*V*_redox_ is from the unequal potential drop at the electrode-solution interface.

The maximum energy conversion efficiency (*η*_max_) can be calculated by the equation as follows [S1, S2, S4]：

$$ŋ_{\max}=\frac{\left( 2t_{+}-1 \right)^{2}}{2} \left( S2 \right)$$

*t_+_* is the cation transference number, which could be calculated as [S1, S2, S4]:

$$t_{+}=\left( \frac{V_{diff}}{\frac{RT}{zF}ln\frac{\gamma_{H}C_{H}}{\gamma_{L}C_{L}}}+1 \right) \left( S3 \right)$$

where R, T, F, z, γ, and C refer to the universal gas constant, temperature, Faraday constant, charge number, activity coefficient of ions, and ion concentration, respectively. When the membrane material is perfectly cation selective, *t*_+_ reaches the maximum value of 1, and the maximum energy conversion efficiency can be calculated as 50% according to the equation.

**S2 Calculation of the channel width**

The width of the nanofluidic channels (*h*) was calculated as [S5, S6]:

$$h=\frac{2\sigma_{S}t_{Na,bulk}}{cN_{A}q} \left( S4 \right)$$

where σ_S_ is the surface charge density, N_A_ is Avogadro’s number, and q is the charge of a single electron.

*t*_Na, bulk_ is the transference number of sodium ions, which could be calculated as [S7]

$$\begin{aligned} t_{Na, bulk}=\frac{\mu_{Na}}{\mu_{Cl}+\mu_{Cl}} \left( S5 \right) \end{aligned}$$

In the tested solutions, *t*_Na, bulk_=0.39. The calculated channel width of PAAS hydrogel is 2.2 nm (Table S6).

**S3** **Numerical simulations**

The ionic concentration profiles of cations and anions in the nanochannels of WPAAS-R membrane was theoretically calculated with COMSOL 5.5 Multiphysics. The Numerical simulation was performed based on coupled Poisson and Nernst-Planck equations. The Nernst-Planck equation defined the flux of each ion species. The ionic flux (*J_i_*) was calculated as [S1, S8, S9]:

$$\begin{aligned} J_{i}=-D_{i}\left( \nabla c_{i}+\frac{z_{i}Fc_{i}}{RT}\nabla\varphi\right)+c_{i}u \left( S6 \right) \end{aligned}$$

where *D_i_* is diffusion coefficient, *c_i_* is the concentration, *z_i_* is the charge of the species i, *F* is the Faraday’s constant, *R* is universal gas constant, *T* is temperature, *φ* is the electrical potential and *u* is fluid velocity. The hydrodynamic effects from the fluid flow are assumed negligible (*u=0*).

The relationship between local electric potential (*φ*) and ion concentration (*c_i_*) is shown by Poisson equation [S1, S8, S9]:

$$\begin{aligned} \nabla^{2}\varphi=-\frac{F}{\varepsilon}\sum_{i} z_{i}c_{i} \left( S7 \right) \end{aligned}$$

where *ε* is permittivity of the fluid.

The ionic current (*I*) across the reservoir and the nanochannel is calculated from [S1, S8, S9]:

$$\begin{aligned} I=\int_{S} F\left( Z_{p}J_{p}+Z_{n}J_{n} \right)\cdot ndS \left( S8 \right) \end{aligned}$$

The electrostatic boundary condition is given by [1, 8]:

$$\begin{aligned} n\cdot\nabla\varphi=\frac{\sigma}{\varepsilon_{0}\varepsilon_{r}} \left( S9 \right) \end{aligned}$$

where *n* is the unit normal vector to the wall surface and *σ* is the surface charge density of the walls.

The system is simplified in a steady state condition, and the ionic flux should meet the time-independent continuity when the system approaches a stationary regime [S1, S8, S9]:

$$\begin{aligned} \nabla\cdot J_{i}=0 \left( S10 \right) \end{aligned}$$

The model of the numerical simulations is shown in Fig. S18. It contains a 30 nm long 2D channel, and the size of the channel were set as 2 nm, 4 nm and 6 nm. To minimize the effect of the resistance of mass transfer at the entrance and exit, two electrolyte reservoirs (20 nm × 10 nm) were added. Under the appropriate boundary conditions, the coupled equations could be solved with finite-element calculations for the ion concentration distribution. NaCl solution was used in the simulation. The concentration gradient is set to 50-fold (i.e. 0.5 M/0.01 M). The surface charge density on the walls of the reservoir was assumed to be zero. The external potential is applied across the channel.

**Supplementary Figures and Tables**


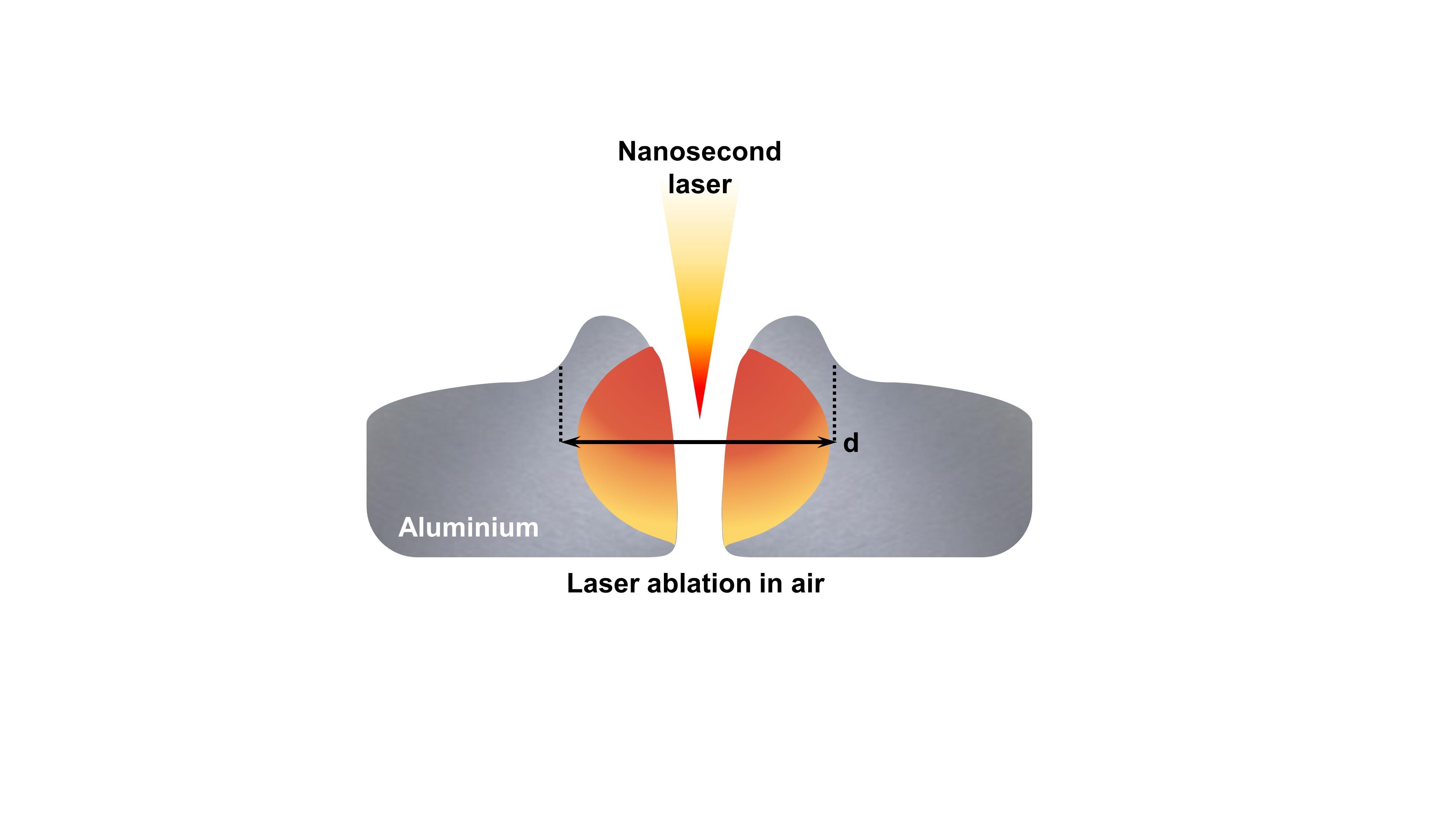


**Fig. S1** Schematic illustration of preparing the submillimeter pore with nanosecond laser


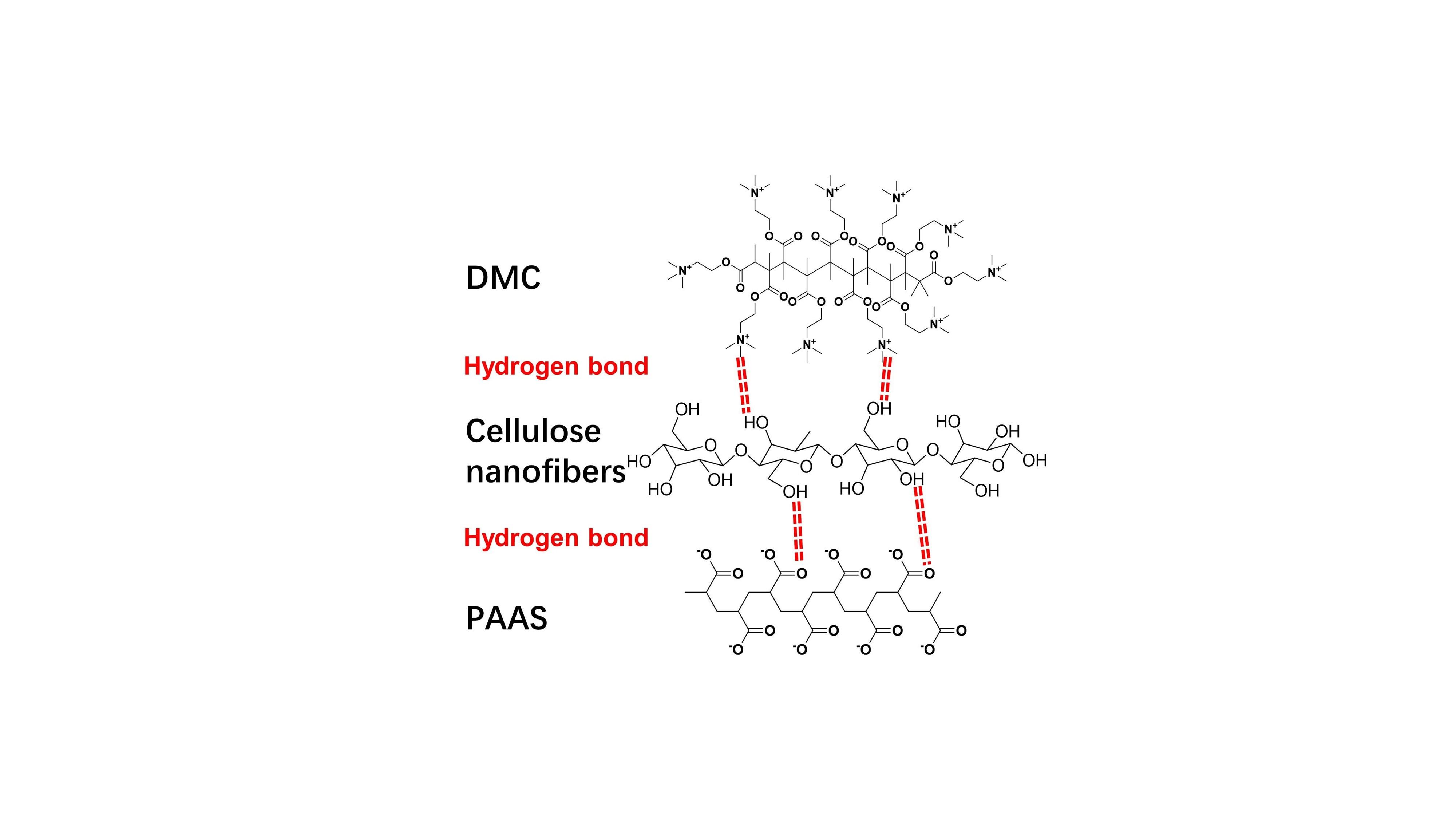


**Fig. S2** Hydrogen bonds are formed between PAAS, DMC and CNFC


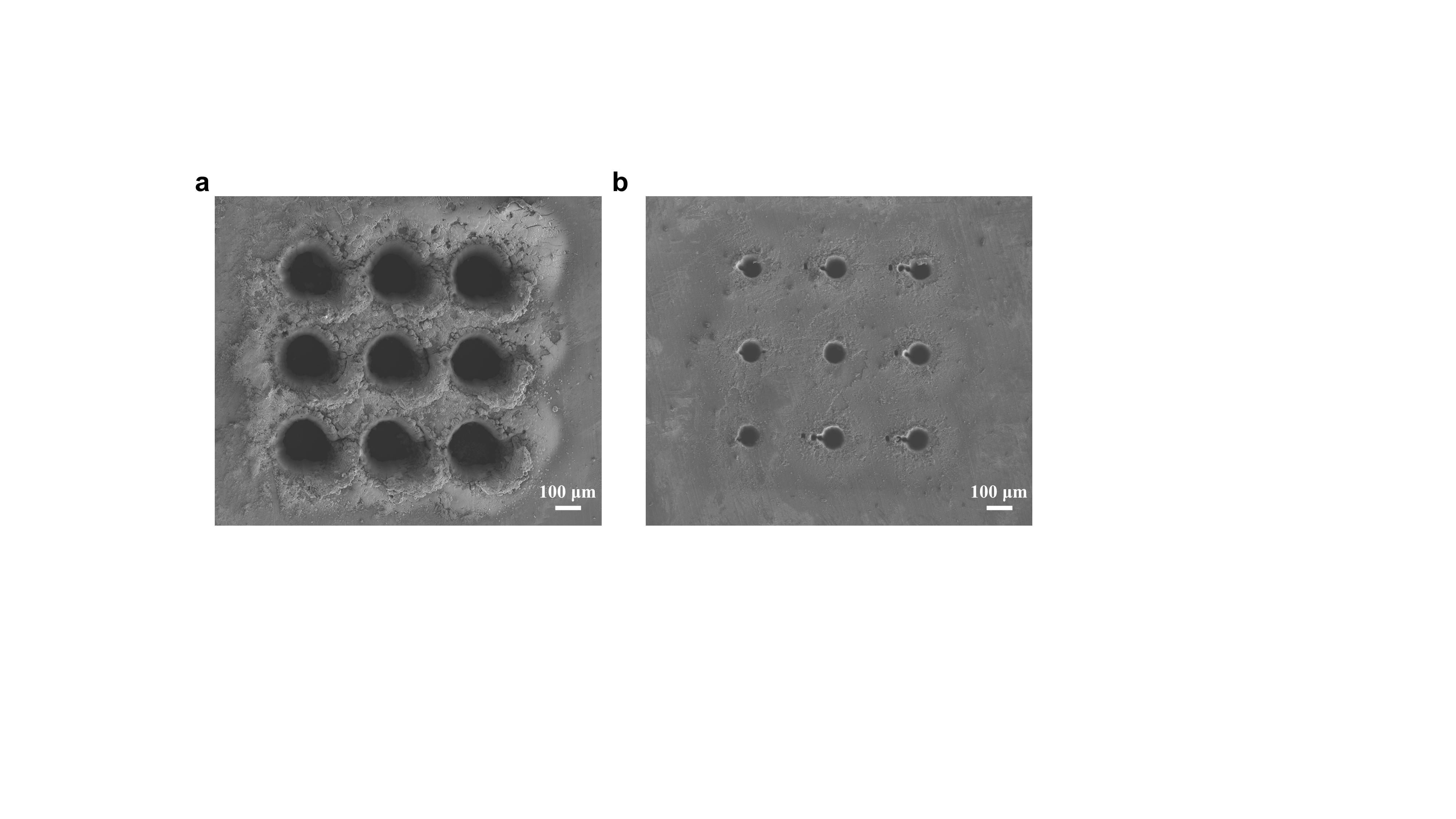


**Fig. S3** SEM images of aluminum plate with 9 pores. **a**) The semi-diameter of the pore is about 0.1 mm. **b**) The semi-diameter of the pore is about 0. 05 mm


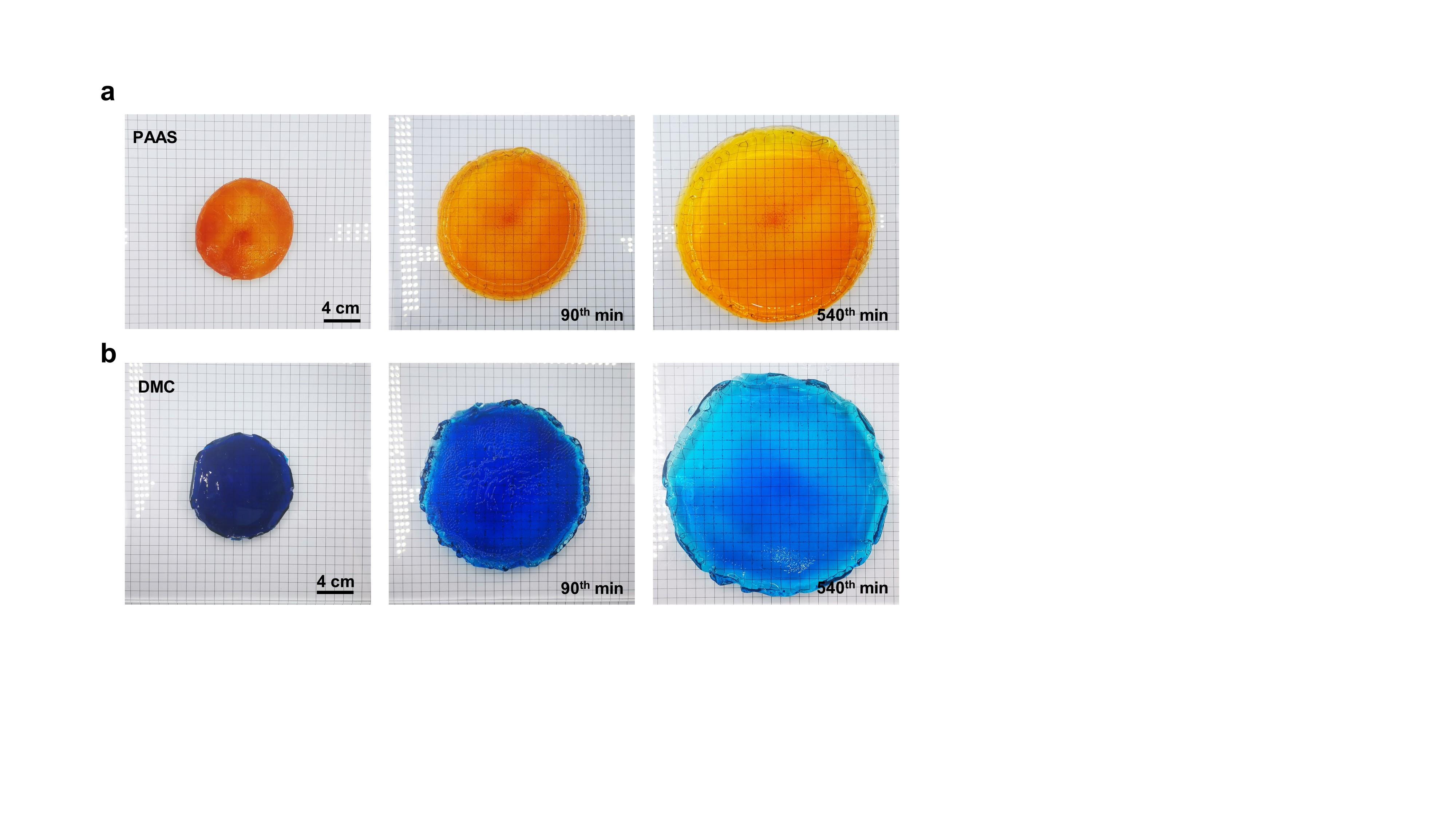


**Fig. S4** The free swelling process of a) PAAS and b) DMC hydrogel in 0.5M NaCl


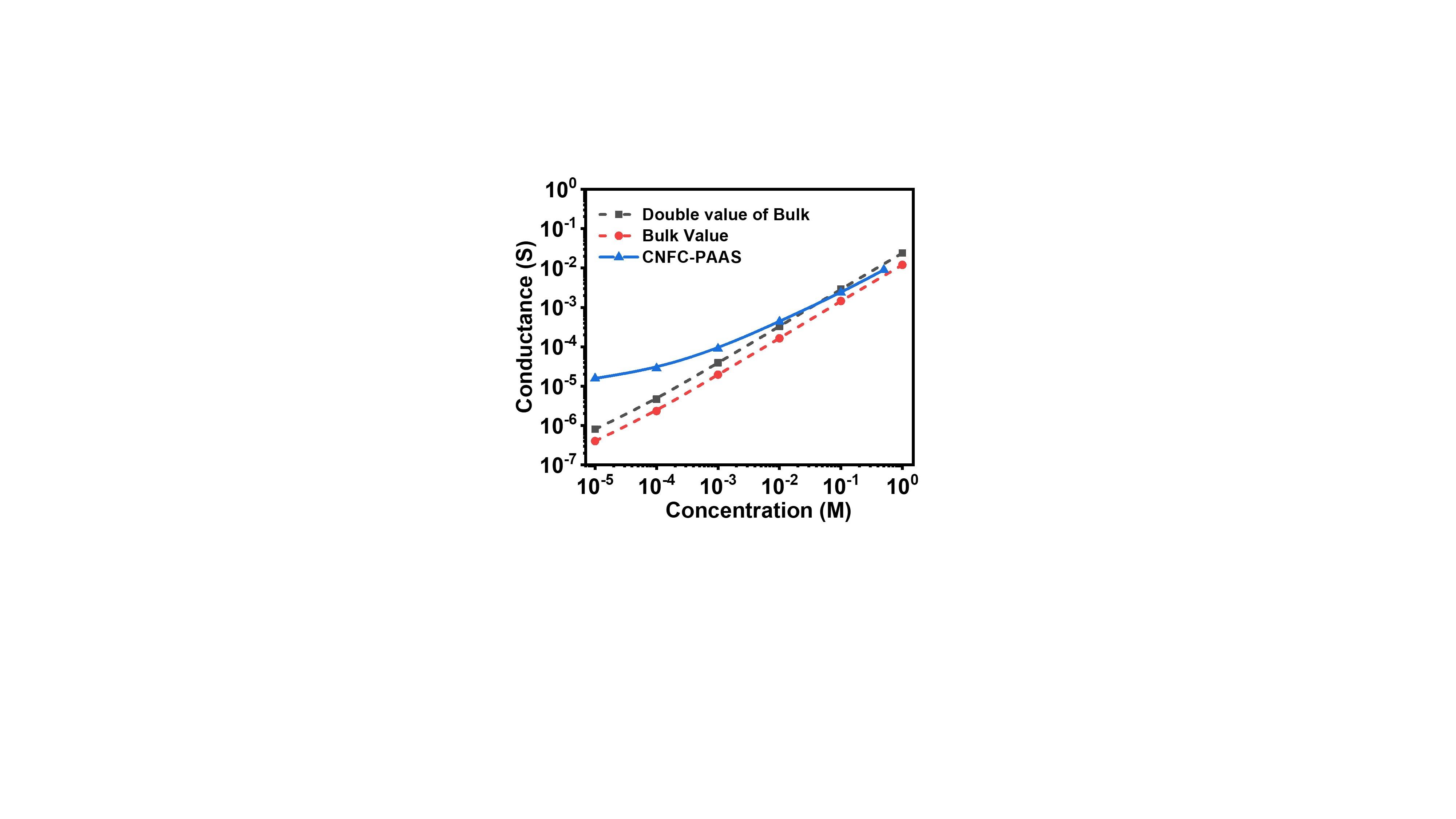


**Fig. S5** Ionic conductivity of the CNFC-PAAS hydrogel at different NaCl concentrations. Blue and black lines represent the CNFC-PAAS hydrogel channel and bulk conductivities, respectively. The green line represents doubled values of bulk conductance. At the concentration where the blue and green lines intersect, the surface charge contribution to conductance is taken to be equal to the bulk contribution.


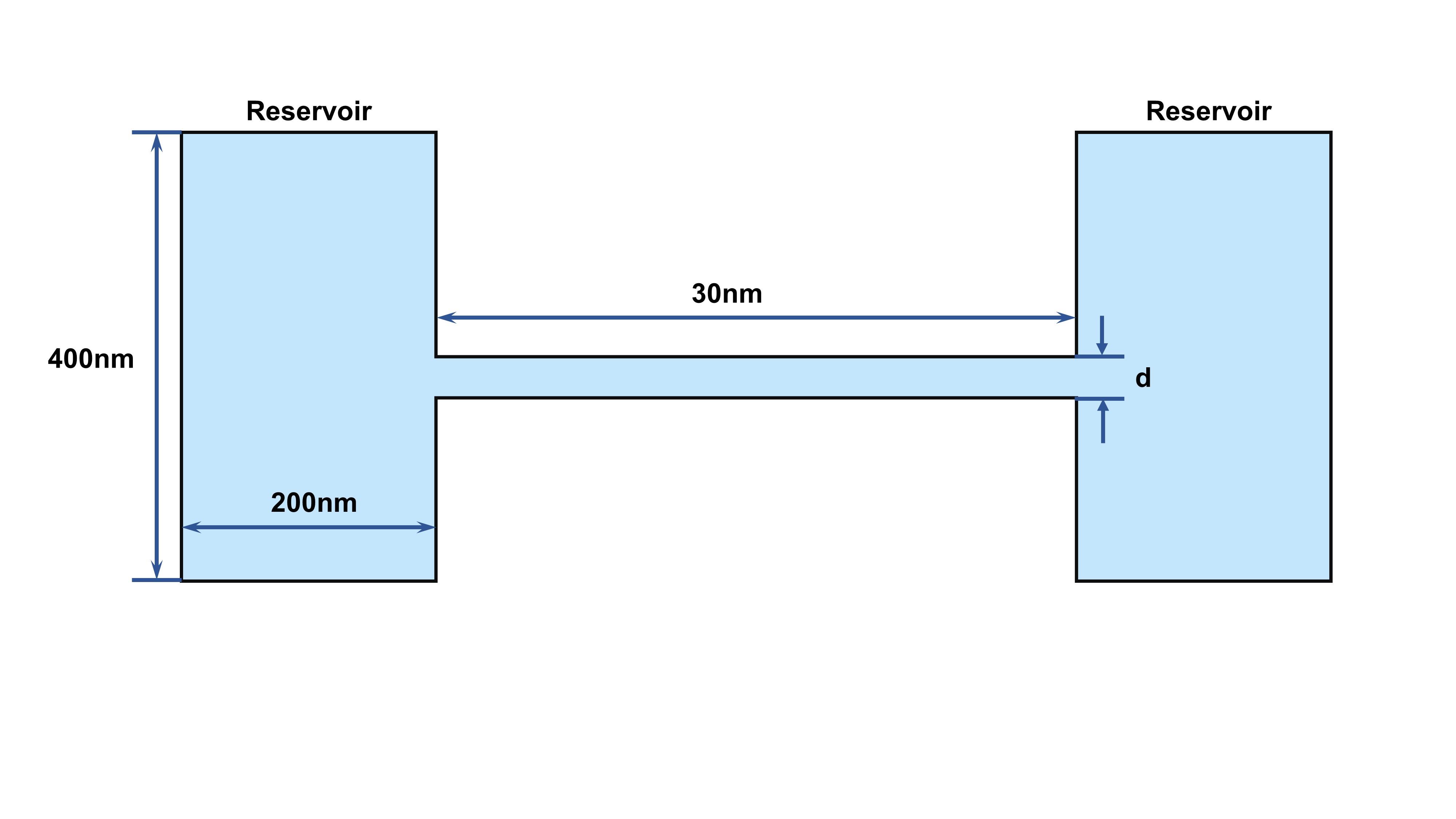


**Fig. S6** Model of theoretical simulation. In this work, the 2D channel with three different distances (including 2 nm, 4 nm and 6 nm) were simulated (Drawing not to scale)


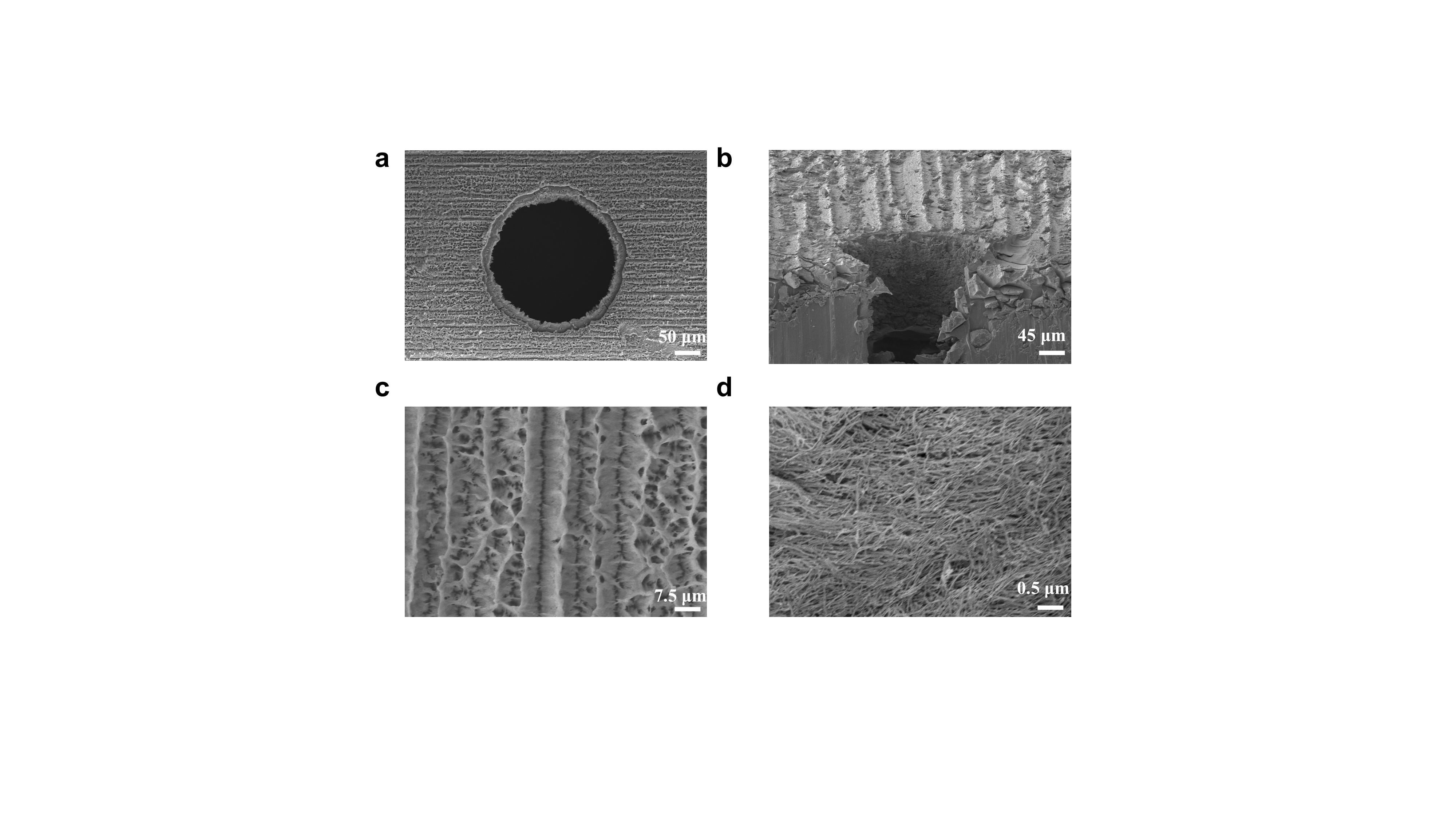


**Fig. S7** **a-b**) SEM image of the surface of AAS with micro pore. **c-d**) SEM image of etching marks on the surface of the aluminum sheet


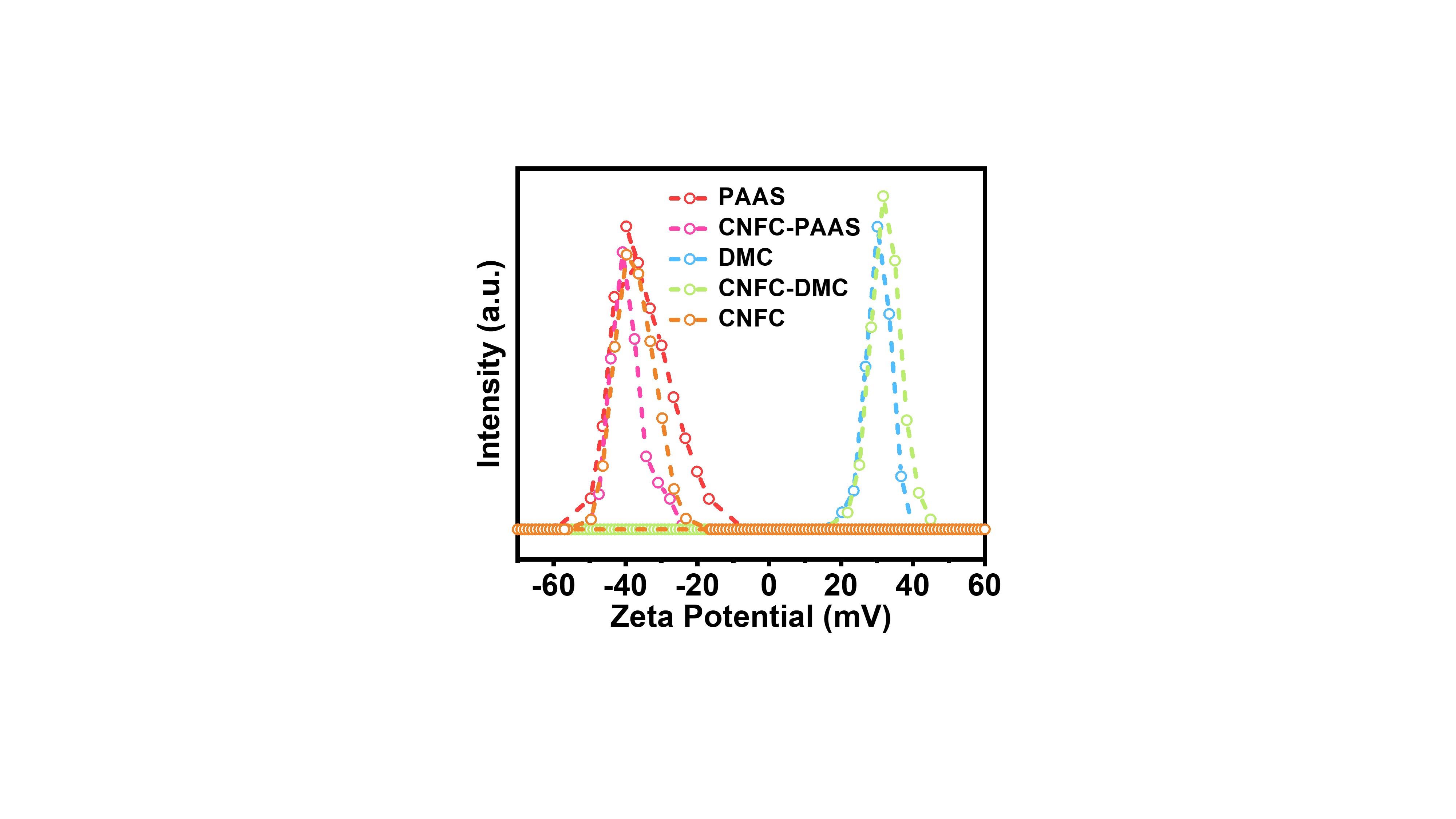


**Fig. S8** Zeta potentials of positively/negatively charged PAAS, CNFC-PAAS, DMC, CNFC-DMC and CNFC


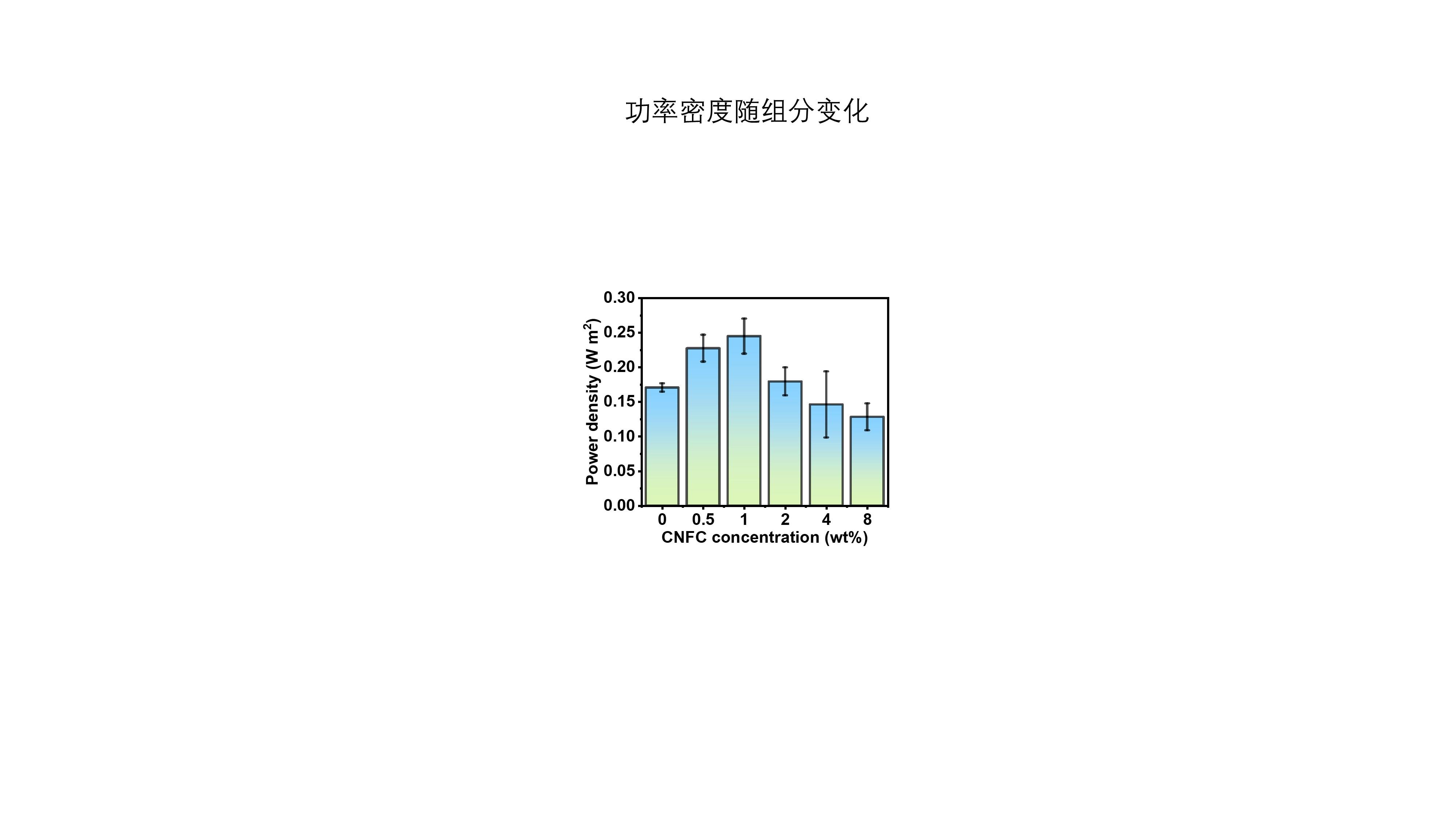


**Fig. S9** Maximum output power density of CNFC-PAAS hydrogel membrane with different CNFC concentration. The test area here is 3.14 mm^2^


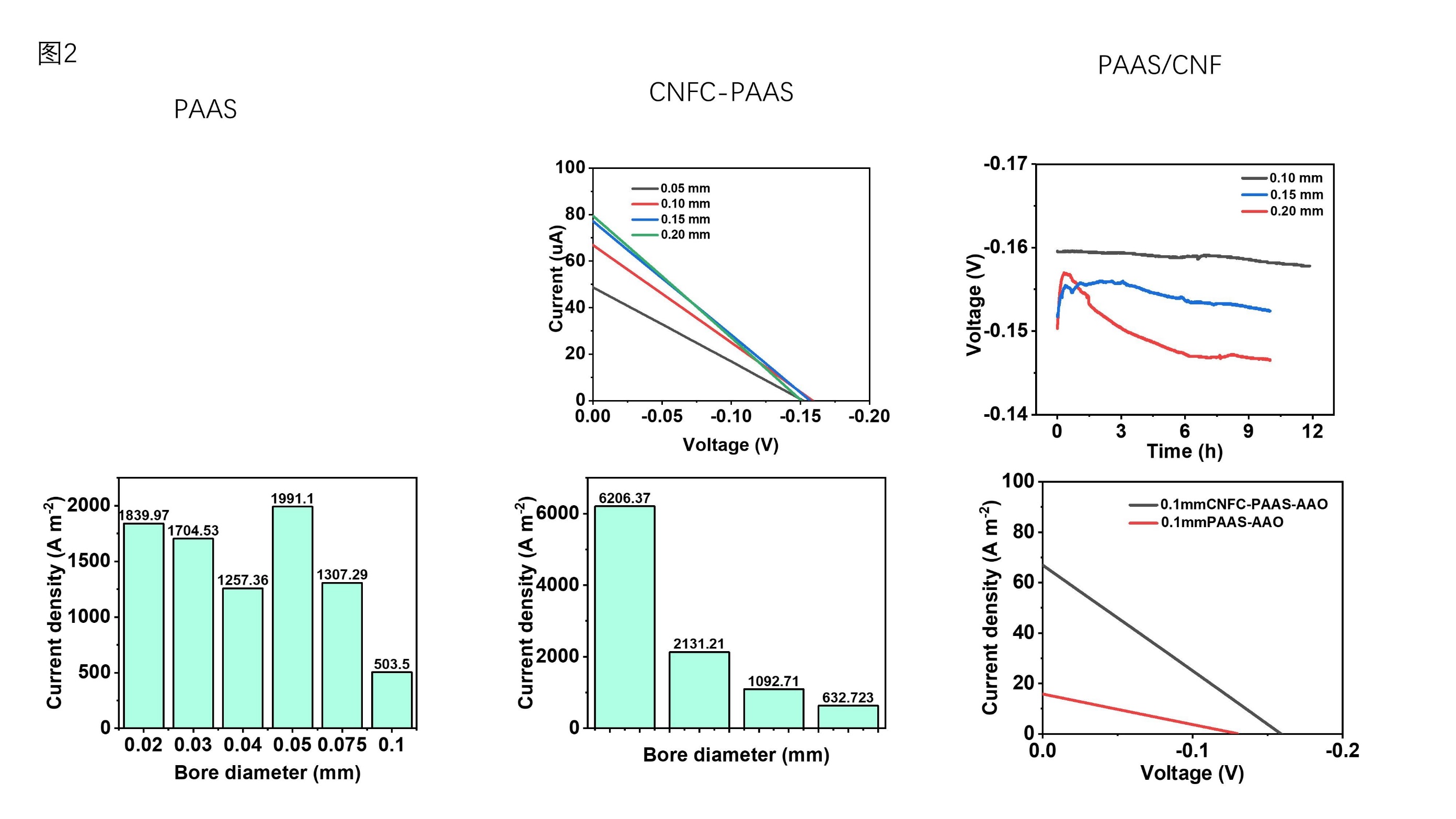


Fig. S10 I-V curves of CNFC-PAAS-AAS membranes with different pore sizes


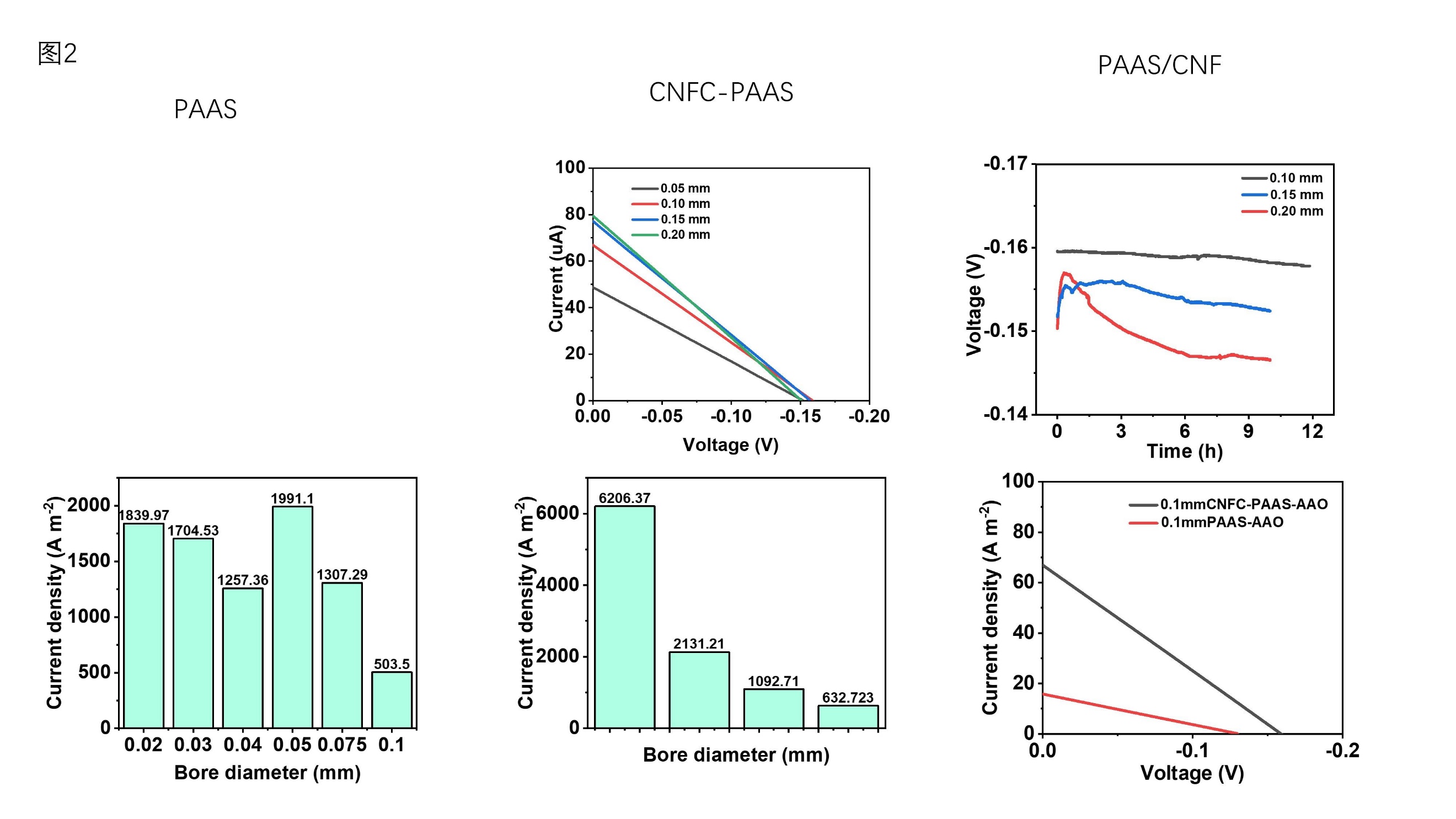


Fig. S11 V-t curves of the CNFC-PAAS-AAS membranes with different pore radius


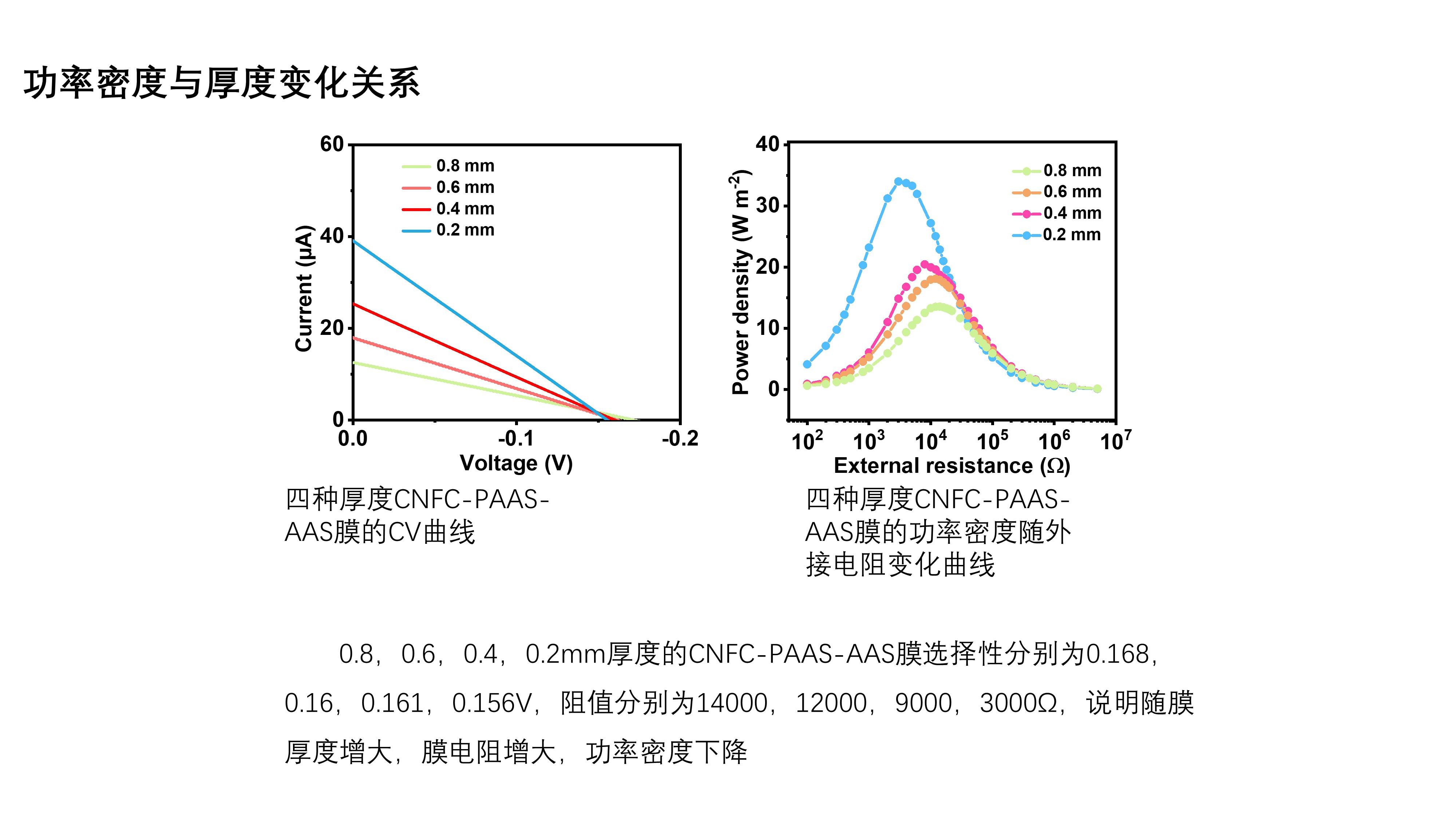


Fig. S12 a I-V curves of CNFC-PAAS-AAS membranes with different thickness. b The output power density of CNFC-PAAS-AAS membranes with different thickness


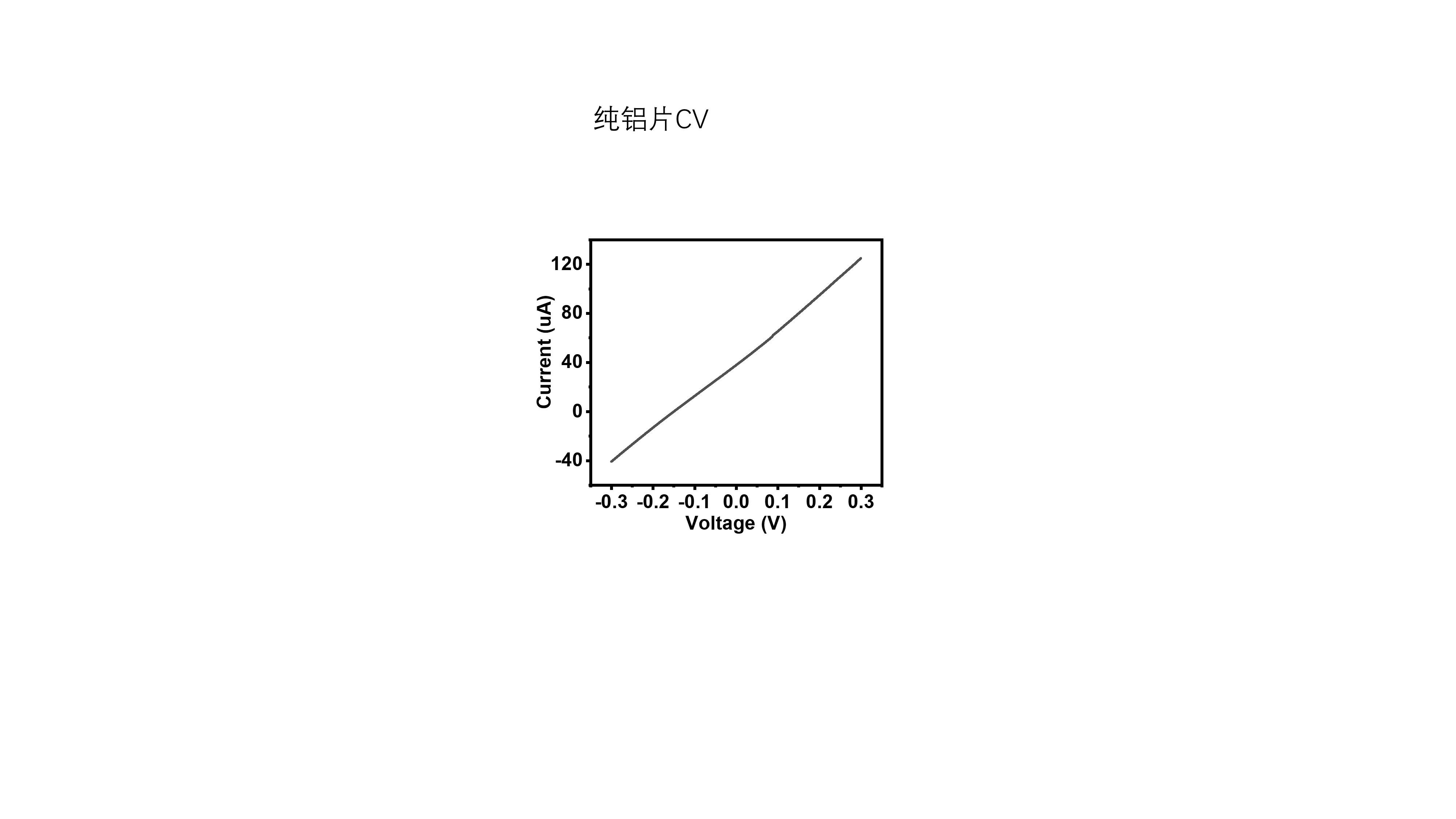


Fig. S13 I-V curves of CNFC-PAAS hydrogel which was confined in neutral aluminium substrate


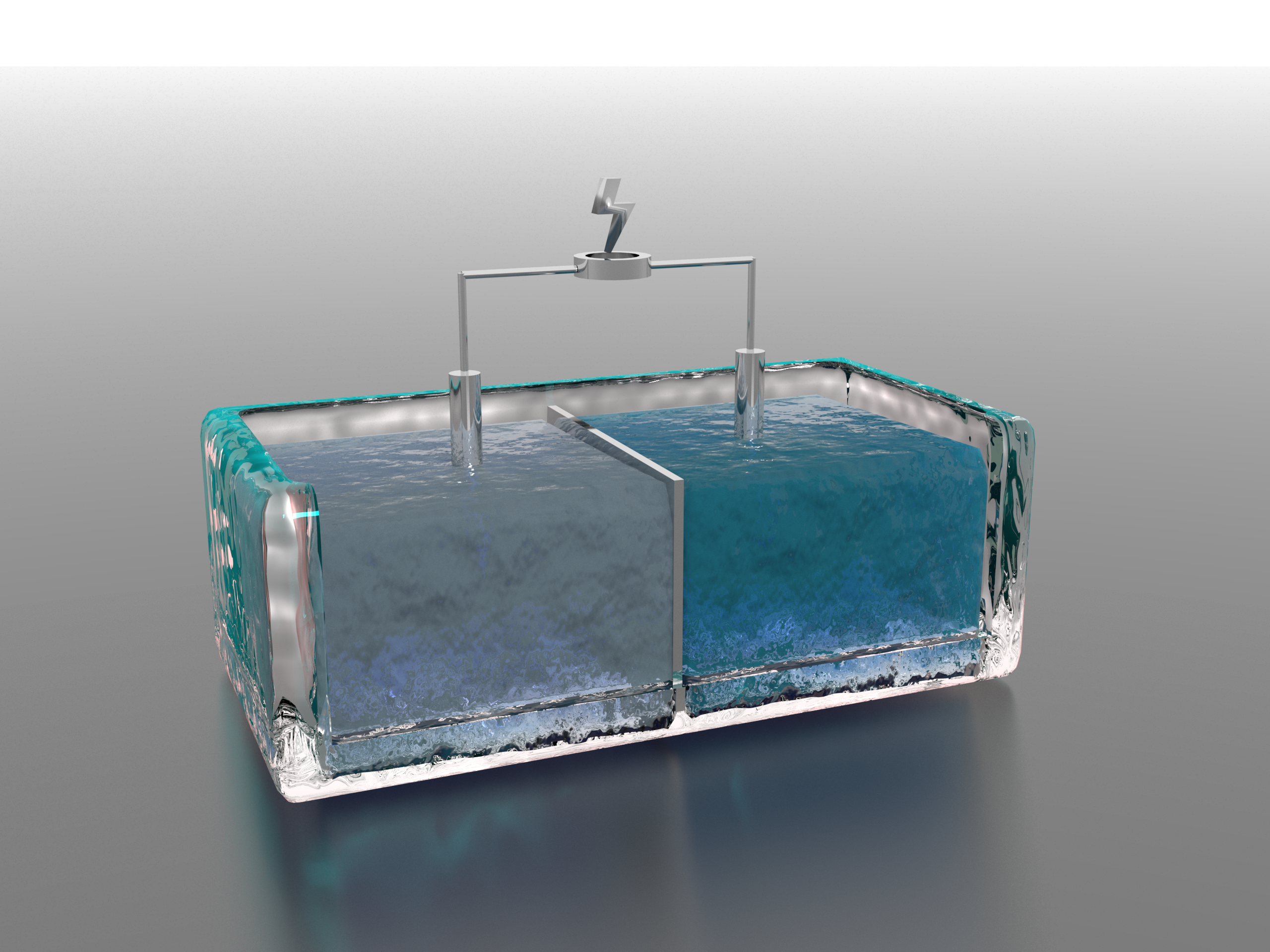


Fig. S14 Schematic diagram of the two-chamber experimental setup


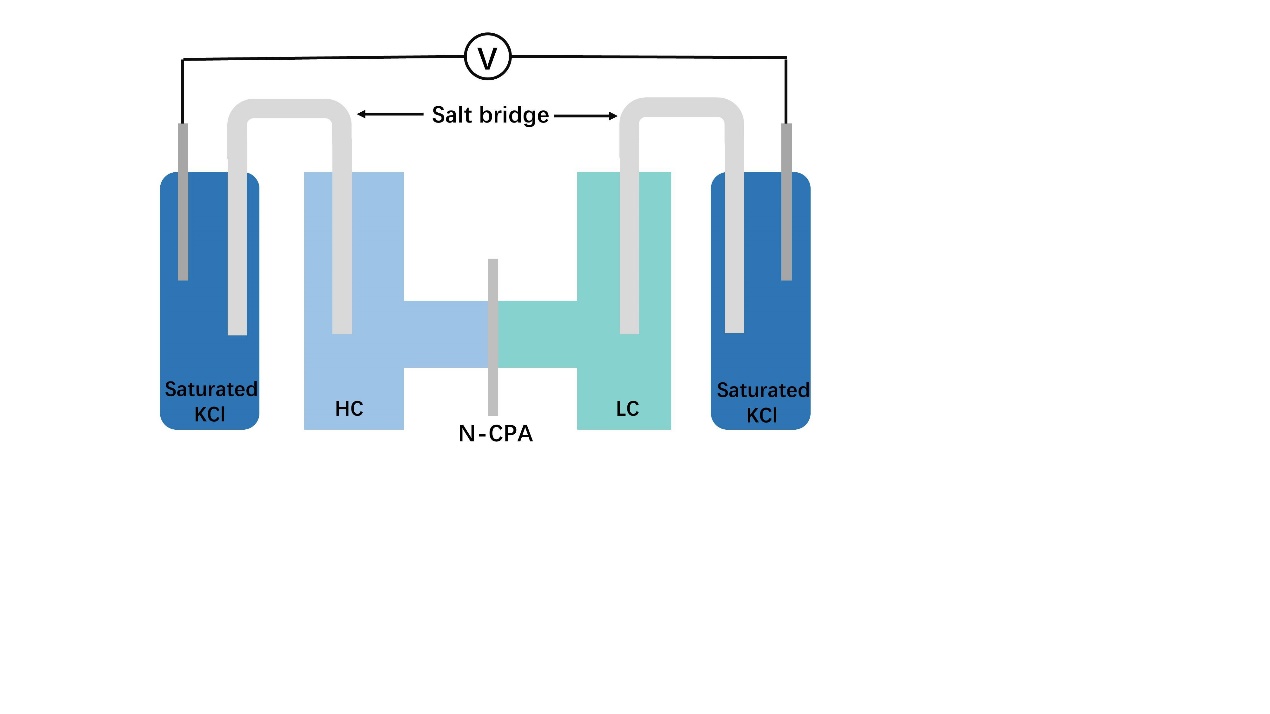


Fig. S15 Schematic diagram of electrochemical cell to eliminate electrode potential


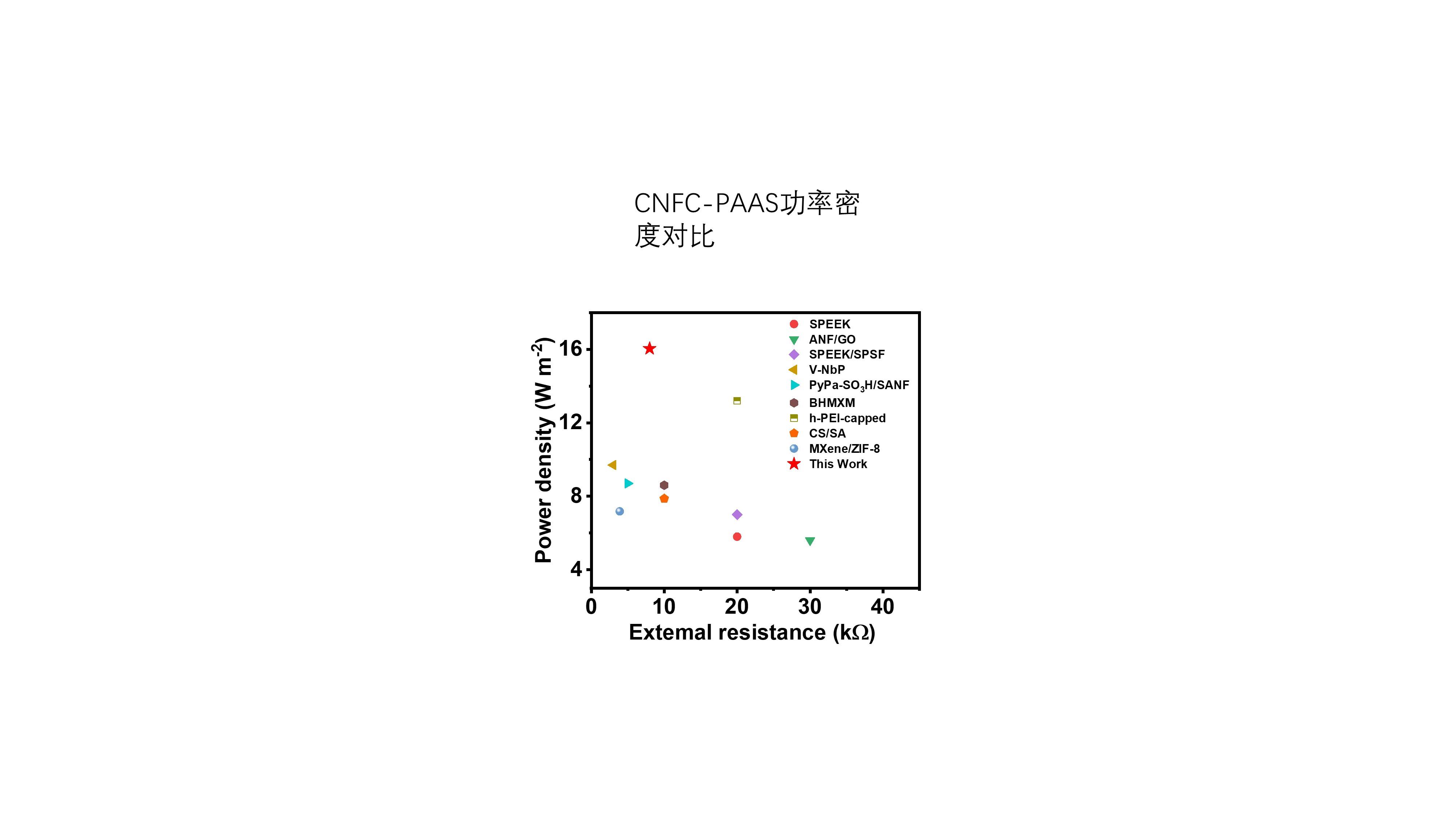


**Fig. S16** Power generation performance of CNFC-PAAS-AAS (red star) compared with state-of-the-art osmotic power generators


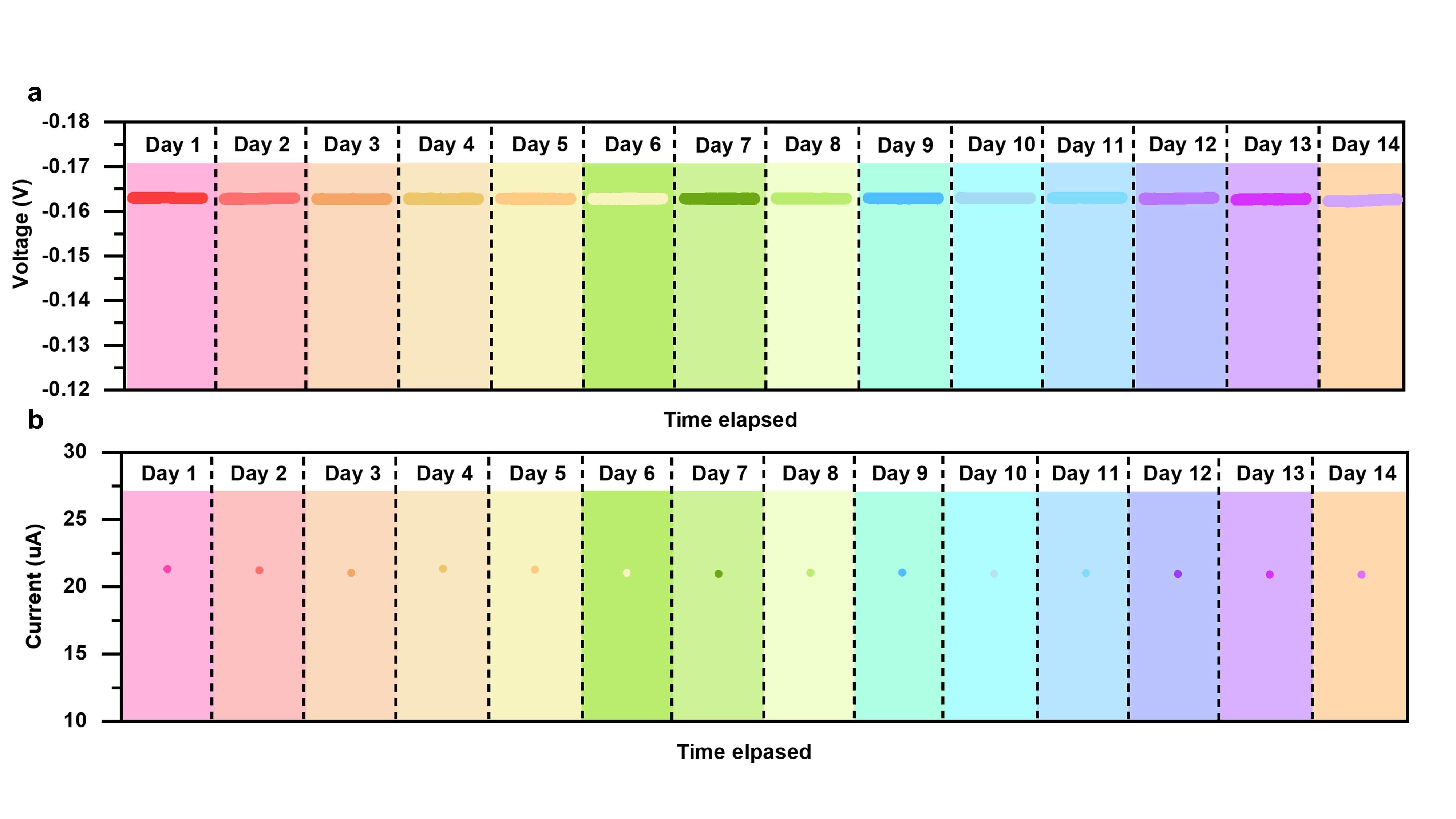


**Fig. S17 a** Open-circuit voltage of CNFC-PAAS-AAS membrane in a continuous 14 days testing. **b** Short-circuit current of CNFC-PAAS-AAS membrane in a continuous 14 days testing


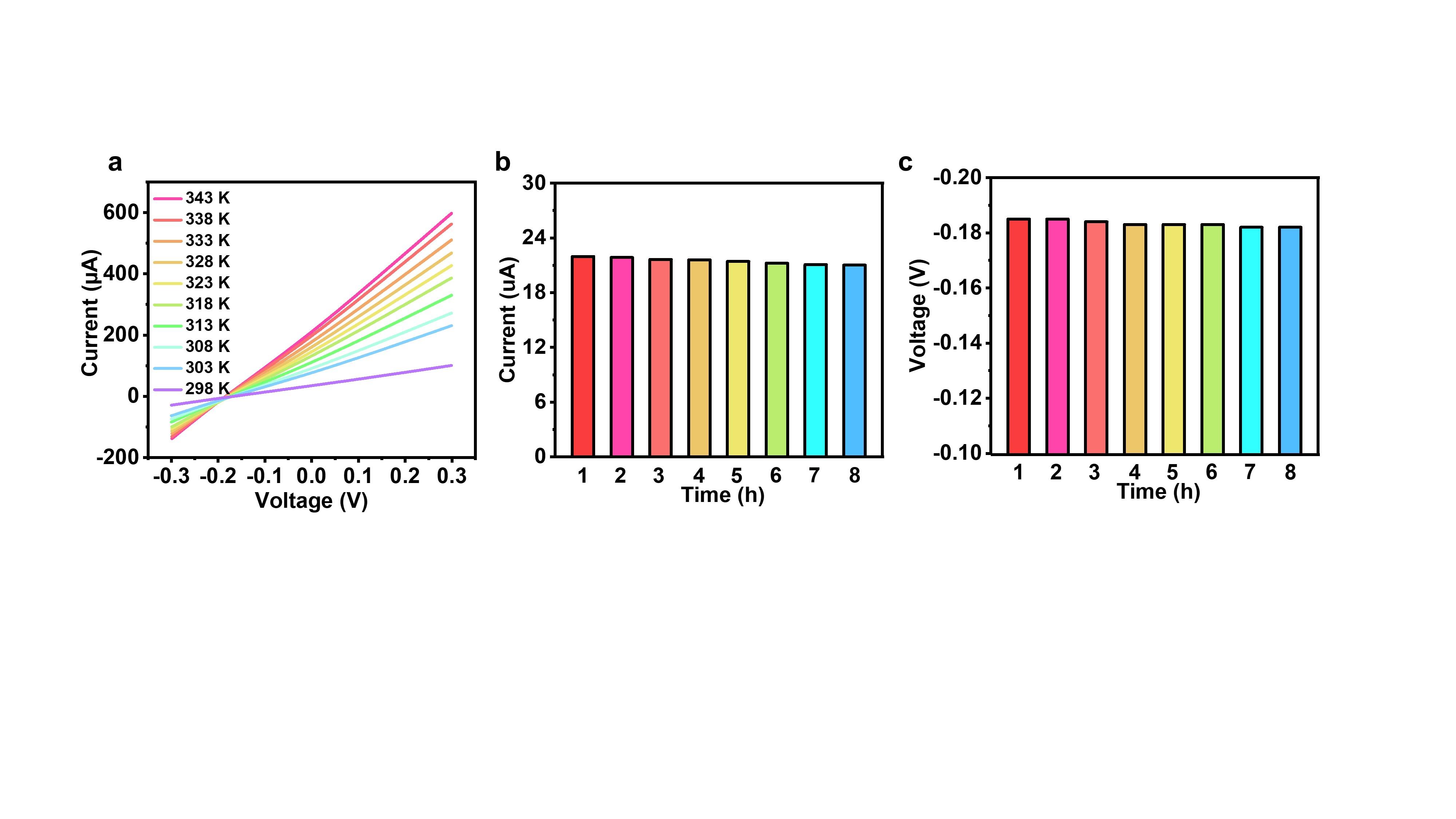


**Fig. S18 a** I-V curves of CNFC-PAAS-AAS membranes confined in different temperature. **b** 8 h current of CNFC-PAAS-AAS membranes at 343 K. **c** 8 h voltage of CNFC-PAAS-AAS membranes at 343 K


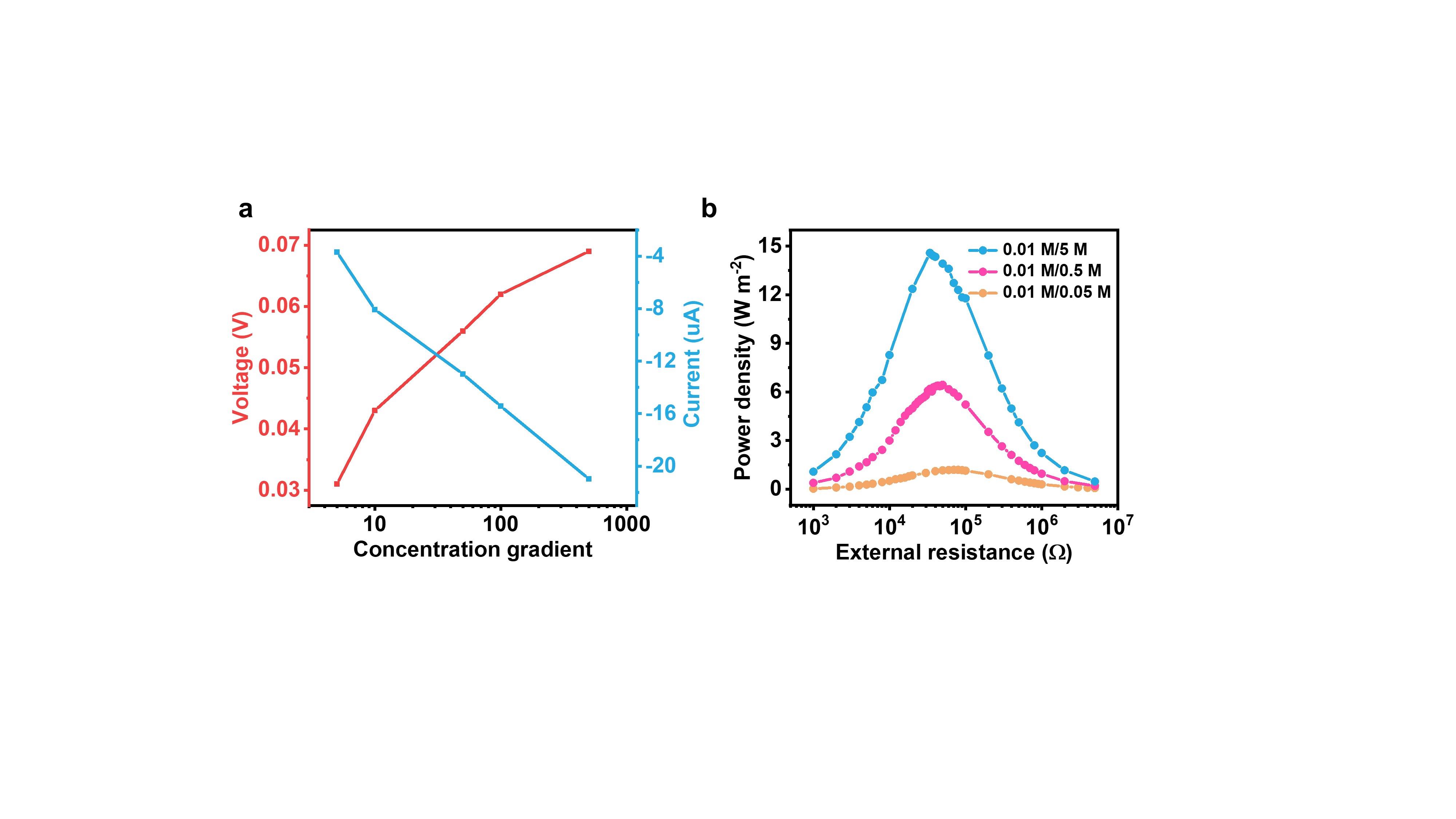


**Fig. S19 a** Osmotic voltage and osmotic current of the CNFC-DMC-AAS membrane as a function of the salt gradient. **b** Output power density of the CNFC-DMC-AAS membrane with different external resistance under three different salinity gradients


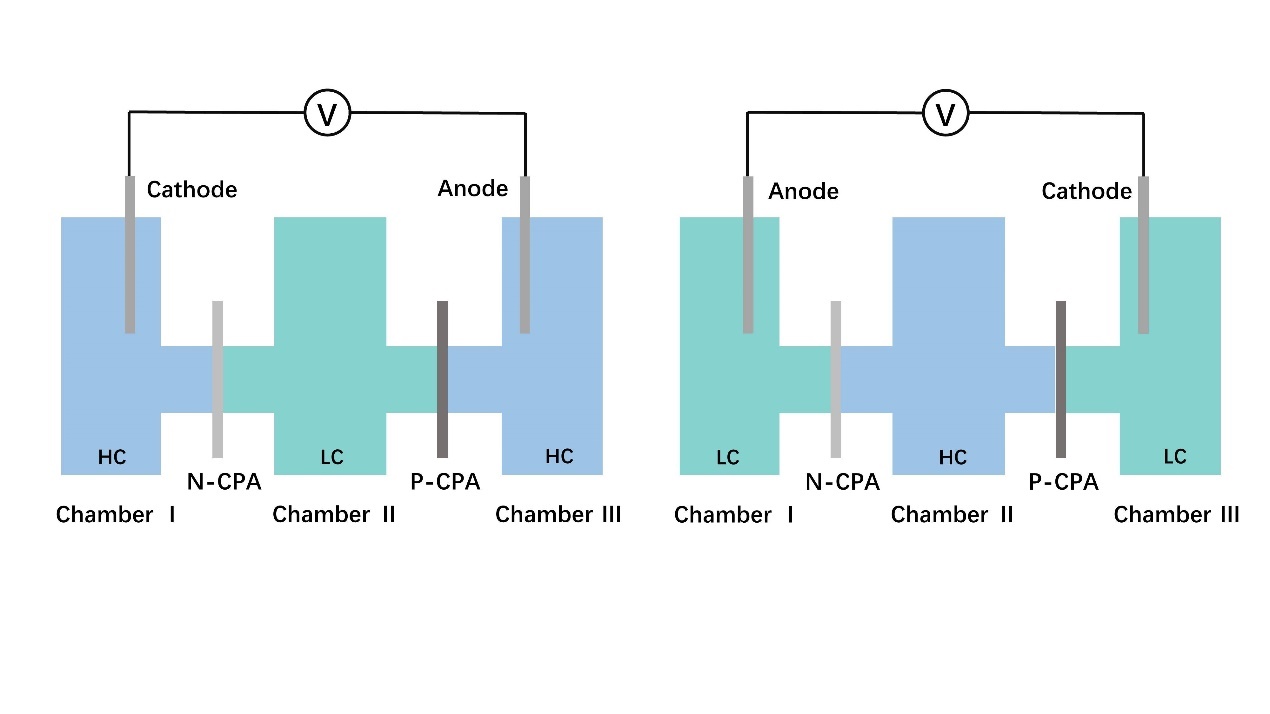


Fig. S20 Three-chamber electrochemical cell consisting of a pair of n-CPA and p-CPA with altering seawater and river water. The capacity of the chamber is 20 mL


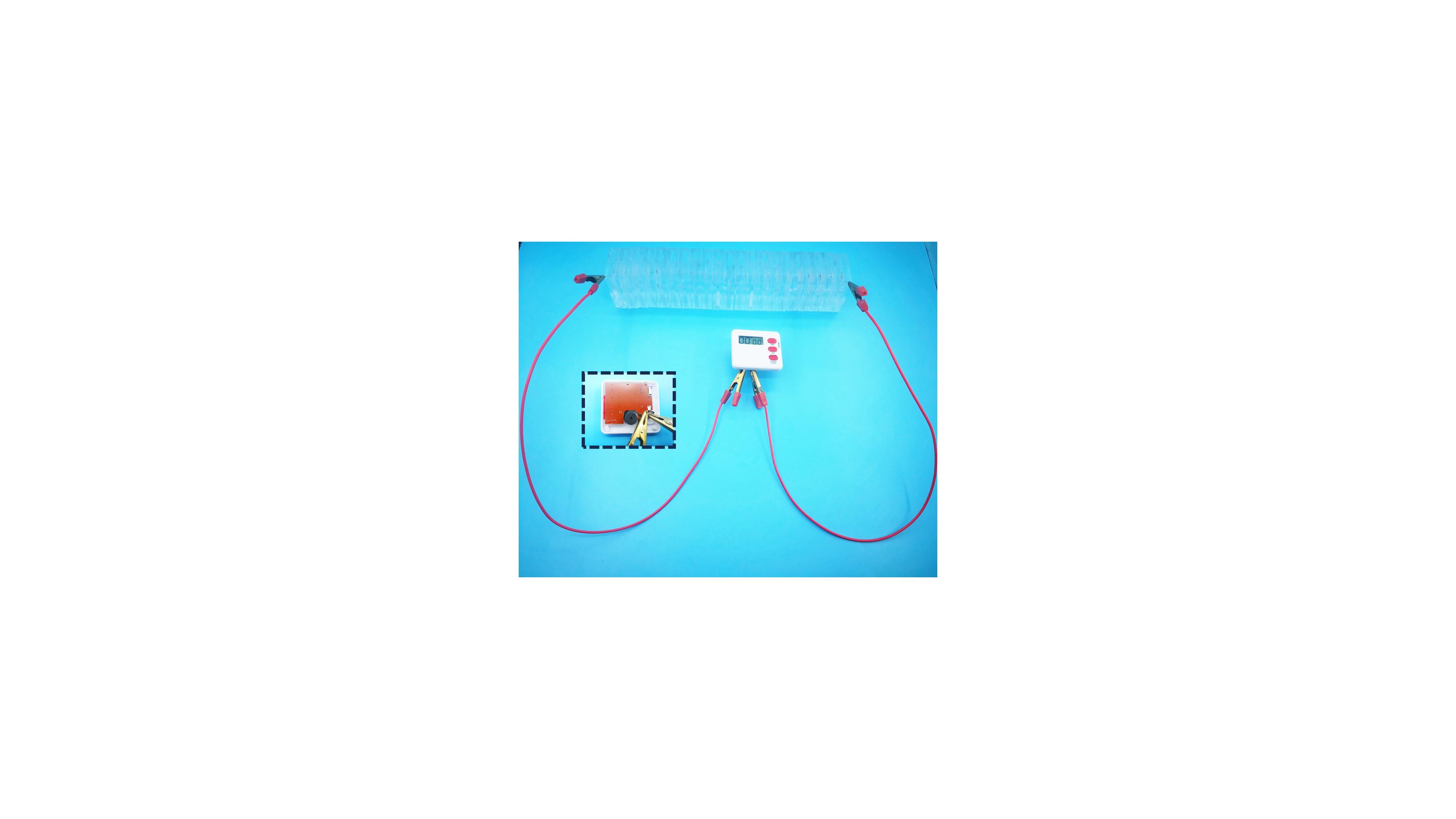


Fig. S21 RED system with serial cell unites can directly power an electronic timer. Insert is the back of the electronic timer

**Table S1** The corresponding *V*_oc_、*V*_redox_、*V*_diff_、*t_+_* and *η* of CNFC-PAAS-AAS membrane

| ***C_High_*/*C_Low_***  **(M/M)** | ***V_oc_***  **(mV)** | ***V_redox_***  **(mV)** | ***V_diff_***  **(mV)** | ***t_+_*** | ***η*** |
| --- | --- | --- | --- | --- | --- |
| 5/0.01 | 239 | 136 | 103 | 0.822 | 0.207 |
| 1/0.01 | 196 | 108 | 88 | 0.896 | 0.313 |
| 0.5/0.01 | 154 | 79 | 75 | 0.899 | 0.318 |
| 0.1/0.01 | 92 | 47 | 45 | 0.901 | 0.321 |
| 0.05/0.01 | 77 | 45 | 32 | 0.912 | 0.336 |

**Table S2** Literature used to compare data in Fig. S14

| Membrane | Resistance (KΩ) | 0.01/0.5M Power density  (W m^-2^) | Test area  (mm^2^) | DOI |
| --- | --- | --- | --- | --- |
| MXene/Kevlar | 27 | 4.1 | 0.0314 | 10.1038/s41467-019-10885-8 |
| SPEEK | 20 | 5.8 | 0.0314 | 10.1093/nsr/nwaa057 |
| GO/CNFS | 30 | 3.8 | 0.0314 | 10.1021/acsami.1c03192 |
| ANF/GO | 30 | 5.6 | 0.0314 | 10.1021/acscentsci.1c00633 |
| SPEEK/SPSF | 20 | 7 | 0.0314 | 10.1016/j.nanoen.2020.105657 |
| V-NbP | 3 | 9.7 | 0.0314 | 10.1021/jacs.2c12936 |
| PyPa-SO_3_H/SANF | 5 | 8.7 | 0.0314 | 10.1021/jacs.1c07392 |
| BHMXM | 10 | 8.6 | 0.0314 | 10.1002/anie.202206152 |
| h-PEI-capped | 20 | 13.2 | 0.0314 | 10.1126/sciadv.abg2183 |
| CS/SA | 10 | 7.87 | 0.0314 | 10.1002/anie.202108549 |
| MXene/ZIF-8 | 3.9 | 7.18 | 0.0314 | 10.1002/adfm.202209767 |
| This Work | 8 | 16.04 | 0.0314 |  |

**Table S3** The corresponding *V*_diff_、***t_+_*** and ***η*** of CNFC-DMC-AAS membrane

| ***C_High_*/*C_Low_***  **(M/M)** | ***V_diff_***  **(mV)** | ***t_+_*** | ***η*** |
| --- | --- | --- | --- |
| 5/0.01 | 69 | 0.281 | 0.096 |
| 1/0.01 | 62 | 0.22 | 0.157 |
| 0.5/0.01 | 56 | 0.202 | 0.177 |
| 0.1/0.01 | 43 | 0.117 | 0.293 |
| 0.05/0.01 | 31 | 0.102 | 0.317 |

**Table S4** The NaCl Concentration of Xiaoqing River and Huanghai Sea

|  | ***NaCl Concentration*** (mol L^-1^) |
| --- | --- |
| Xiaoqing River | 0.0073 |
| Huanghai Sea | 0.504 |

**Table S** Literature used to compare data in Fig. 6f

| Membrane | Resistance (KΩ) | 0.01/0.5M Power density  (W m^-2^) | DOI |
| --- | --- | --- | --- |
| ChCNC/GO | 7 | 4.73 | [10.1016/j.nanoen.2022.107291](https://www.x-mol.com/paperRedirect/1516624512963485696) |
| RC/CNTs | 25 | 5.28 | [10.1016/j.nanoen.2023.108693](https://www.x-mol.com/paperRedirect/1679705407540150272) |
| MXM-RED | 5 | 4.6 | [10.1002/anie.201915993](https://www.x-mol.com/paperRedirect/1218451572886884352) |
| GO | 10 | 0.77 | [10.1002/adfm.201603623](https://www.x-mol.com/paperRedirect/180973) |
| MXene | 27 | 3.7 | [10.1038/s41467-019-10885-8](https://www.x-mol.com/paperRedirect/5751682) |
| BCM-RED | 100 | 0.72 | [10.1016/j.nanoen.2022.107786](https://www.x-mol.com/paperRedirect/1568068758461976576) |
| BCC-pair RED | 155 | 0.7 | [10.1016/j.nanoen.2022.107548](https://www.x-mol.com/paperRedirect/1541857206948958208) |
| MF-RED | 0.5 | 12.3 | [10.1039/d2ta06557f](https://www.x-mol.com/paperRedirect/1589662361207668736) |
| wood-based RED | 9 | 5.14 | [10.1002/aenm.201902590](https://www.x-mol.com/paperRedirect/5961899) |
| CNFC-PAAS/DMC-AAS RED  (This work) | 8 | 8.99 | This work |

**Table S6** The width of the nanofluidic channels (*h*) and the surface charge density (σ_S_) of confined CNFC-PAAS and CNFC-DMC

|  | ***h* (nm)** | ***σ_S_* (C m^-2^)** |
| --- | --- | --- |
| CNFC-PAAS | 2.18 | -0.0108 |
| CNFC-DMC | 1.96 | 0.0097 |

**Supplementary References**

1. Sheng N., Zhang M., Song Q., Zhang H., Chen S., et al., Enhanced salinity gradient energy harvesting with oppositely charged bacterial cellulose-based composite membranes. Nano Energy **101**, 107548 (2022). <http://dx.doi.org/10.1016/j.nanoen.2022.107548>
2. Chen J., Xin W., Chen W., Zhao X., Qian Y., et al., Biomimetic Nanocomposite Membranes with Ultrahigh Ion Selectivity for Osmotic Power Conversion. ACS Cent. Sci. **7**, 1486-1492 (2021). <http://dx.doi.org/10.1021/acscentsci.1c00633>
3. Hu Y., Teng Y., Sun Y., Liu P., Fu L., et al., Bioinspired poly (ionic liquid) membrane for efficient salinity gradient energy harvesting: Electrostatic crosslinking induced hierarchical nanoporous network. Nano Energy **97**, 107170 (2022). <http://dx.doi.org/10.1016/j.nanoen.2022.107170>
4. Wu Y., Xin W., Kong X.-Y., Chen J., Qian Y., et al., Enhanced ion transport by graphene oxide/cellulose nanofibers assembled membranes for high-performance osmotic energy harvesting. Mater. Horizons **7**, 2702-2709 (2020). <http://dx.doi.org/10.1039/d0mh00979b>
5. Wu Q.Y., Wang C., Wang R., Chen C., Gao J., et al., Salinity-Gradient Power Generation with Ionized Wood Membranes. Adv. Energy Mater. **10**, 1902590 (2019). <http://dx.doi.org/10.1002/aenm.201902590>
6. Shao J.-J., Raidongia K., Koltonow A.R., Huang J. Self-assembled two-dimensional nanofluidic proton channels with high thermal stability. Nat. Commun. **6**, 7602 (2015). <http://dx.doi.org/10.1038/ncomms8602>
7. Lao J., Lv R., Gao J., Wang A., Wu J., et al., Aqueous Stable Ti_3_C_2_ MXene Membrane with Fast and Photoswitchable Nanofluidic Transport. ACS Nano **12**, 12464-12471 (2018). <http://dx.doi.org/10.1021/acsnano.8b06708>
8. Qin R., Tang J., Wu C., Zhang Q., Xiao T., et al., Nanofiber-reinforced clay-based 2D nanofluidics for highly efficient osmotic energy harvesting. Nano Energy **100**, 107526 (2022). <http://dx.doi.org/10.1016/j.nanoen.2022.107526>
9. Ding L., Xiao D., Lu Z., Deng J., Wei Y., et al., Oppositely Charged Ti_3_C_2_T_x_ MXene Membranes with 2D Nanofluidic Channels for Osmotic Energy Harvesting. Angew. Chem. Int. Ed. **59**, 8720-8726 (2020). <http://dx.doi.org/10.1002/anie.201915993>
